# Isolated face features are sufficient to elicit ultra-rapid and involuntary orienting responses toward faces

**DOI:** 10.1167/jov.21.2.4

**Published:** 2021-02-05

**Authors:** Louise Kauffmann, Sarah Khazaz, Carole Peyrin, Nathalie Guyader

**Affiliations:** 1 CNRS, LPNC, University of Grenoble Alpes, University of Savoie Mont Blanc, Grenoble, France; 2 CNRS, Grenoble INP, GIPSA-lab, University of Grenoble Alpes, Grenoble, France; 3 CNRS, LPNC, University of Grenoble Alpes, University of Savoie Mont Blanc, Grenoble, France; 4 CNRS, LPNC, University of Grenoble Alpes, University of Savoie Mont Blanc, Grenoble, France; 5 CNRS, Grenoble INP, GIPSA-lab, University of Grenoble Alpes, Grenoble, France

**Keywords:** eye-tracking, saccadic choice task, face perception, holistic processing

## Abstract

Previous studies have shown that face stimuli influence the programming of eye movements by eliciting involuntary and extremely fast saccades toward them. The present study examined whether holistic processing of faces mediates these effects. We used a saccadic choice task in which participants were presented simultaneously with two images and had to perform a saccade toward the one containing a target stimulus (e.g., a face). Across three experiments, stimuli were altered via upside-down inversion (Experiment 1) or scrambling of thumbnails within the images (Experiments 2 and 3) in order to disrupt holistic processing. We found that disruption of holistic processing only had a limited impact on the latency of saccades toward face targets, which remained extremely short (minimum saccadic reaction times of only ∼120–130 ms), and did not affect the proportion of error saccades toward face distractors that captured attention more than other distractor categories. It, however, resulted in increasing error rate of saccades toward face targets. These results suggest that the processing of isolated face features is sufficient to elicit extremely fast and involuntary saccadic responses toward them. Holistic representations of faces may, however, be used as a search template to accurately detect faces.

## Introduction

The human visual system has evolved to rapidly detect and preferentially process socially relevant stimuli such as faces. For example, face stimuli presented among nonface distractors immediately “pop out,” irrespective of the number of distractors (Hershler & Hochstein, 2005; VanRullen, 2006). Furthermore, they capture and retain attention more than other (in)animate objects, even when they are irrelevant to the task at hand (Ariga & Arihara, 2018; Bindemann, Burton, Hooge, Jenkins, & de Haan, 2005; Devue, Belopolsky, & Theeuwes, 2012; Langton, Law, Burton, & Schweinberger, 2008; Sato & Kawahara, 2015; Simpson, Husband, Yee, Fullerton, & Jakobsen, 2014; Theeuwes & Van der Stigchel, 2006). Using eye-tracking, it has also been shown that when present in visual scenes, faces immediately attract the gaze of observers, who spend most of the exploration time looking at them (Cerf, Paxon Frady, & Koch, 2009; Coutrot & Guyader, 2014; Foulsham, Cheng, Tracy, Henrich, & Kingstone, 2010; Hirvenkari et al., 2013; Marat, Rahman, Pellerin, Guyader, & Houzet, 2013; Judd, Ehinger, Durand, & Torralba, 2009).

Recent studies have also used eye-tracking to investigate the speed of face processing using a saccadic choice task (Boucart et al., 2016; Crouzet, Kirchner, & Thorpe, 2010; Crouzet & Thorpe, 2011; Guyader, Chauvin, Boucart, & Peyrin, 2017; Kauffmann et al., 2019). In this task, two images are simultaneously presented to the observer on the left and right side of the screen. One image contains a target stimulus (e.g., a human face), and the other one contains a distractor (e.g., a vehicle). Participants are asked to perform a saccadic eye movement as fast and accurately as possible toward the image containing the target. Using this task, it has been consistently shown that participants are able to initiate ultra-rapid saccadic responses toward face targets, with minimum saccadic reaction times of only 100–120 ms, whereas longer latencies are observed when the target is of another category (e.g., a vehicle; minimum saccadic reaction times around 140–150 ms; e.g., Crouzet et al., 2010; Guyader et al., 2017, Kauffmann et al., 2019). The latencies of saccades toward face targets are comparable to the short latencies of saccades elicited by the appearance of a dot flashed in the periphery during prosaccade tasks (Fischer & Weber, 1993) and are barely above the earliest responses observed in the visual areas of the ventral stream involved in visual recognition (see Crouzet et al., 2010, for more details). These results therefore highlight the remarkable speed of face detection. This bias for face targets during the saccadic choice task has been replicated across many studies and appears to be very robust as it persists irrespective of experimental factors such as the category of the distractor (e.g., vehicles, animals, butterflies; Boucart et al., 2016; Crouzet et al., 2010; Guyader et al., 2017; Nakano et al., 2013) or the viewing conditions of stimuli (e.g., color, gray-scaling, spatial frequency content, eccentricity; Boucart et al., 2016; Guyader et al., 2017).

Another important finding reported in these studies is that participants were more accurate when the target was a face than when the target belonged to another object category. Also, participants tended to make more error saccades when the distractors were faces than when the distractors were other objects (e.g., vehicle; Crouzet et al., 2010; Guyader et al., 2017; Kauffmann et al., 2019). These findings are consistent with previous studies showing that faces can be more easily detected but also capture and retain attention more than other categories of objects (Ariga & Arihara, 2018; Bindemann et al., 2005; Devue et al., 2012; Langton et al., 2008; Sato & Kawahara, 2015; Simpson, Husband, Yee, Fullerton, & Jakobsen, 2014; Theeuwes & Van der Stigchel, 2006) by eliciting involuntary orienting responses toward them that cannot be easily inhibited (Gilchrist & Proske, 2006; Morand, Grosbras, Caldara, & Harvey, 2010). In a recent study (Kauffmann et al., 2019), we also examined the amplitude of saccades, that is, the distance between the saccade starting and ending points during a saccadic choice task. The amplitude of a saccade is thought to be programmed at its onset and not to be influenced by new visual information once initiated, thereby informing about saccade programming prior to its execution. We observed that saccades toward face targets were larger than saccades toward vehicle targets. This effect persisted even if participants were explicitly instructed to perform their saccade toward a cross added at the center of lateral images, suggesting that saccades toward vehicles tended to be hypometric (i.e., had a shorter amplitude than the one needed to reach the target cross). This result was interpreted in the framework of competitive interactions between saccade programs developing in parallel on a common saccade map (one toward each image; Findlay & Walker, 1999; Godijn & Theeuwes, 2002; Massen, 2004; McPeek et al., 2000; McPeek & Keller, 2002; Mokler & Fischer, 1999; Ramakrishnan et al., 2010; Walker & McSorley, 2006) as reflecting inhibitory influence from the saccade program toward the face stimulus on the saccade program toward the vehicle stimulus, resulting in a reduced amplitude of saccades toward vehicle targets.

Overall, previous eye-tracking data suggest that faces contain specific information that influences the programming of saccades by triggering extremely fast and involuntary orienting responses toward them, while inhibiting competing saccade programs toward other stimuli. However, the nature of the visual information rapidly extracted from face stimuli that could underlie these effects has not been extensively investigated. A large body of literature on face perception indicates that with respect to other categories of visual stimuli, faces are processed and represented holistically: The facial features and their configuration (e.g., two eyes above a nose and above a mouth) are extracted as a whole, rather than independently from each other (Farah, Wilson, Drain, & Tanaka, 1998; Maurer, Le Grand, & Mondloch, 2002; Piepers & Robbins, 2012; Tanaka & Simonyi, 2016). This has been notably illustrated by the fact that face processing is particularly sensitive to any stimulus manipulations that disrupt this interdependent integration of facial features. For example, inversion of faces via a 180° rotation impairs their detection in visual scenes (Lewis & Edmonds, 2003; Rousselet, Macé, & Fabre-Thorpe, 2003). Furthermore, while stimulus inversion generally impairs the processing of any object, this effect has been shown to be larger for face stimuli (Yin, 1969). Disruption of face processing due to face inversion is also supported by electrophysiological and neuroimaging studies showing that face-specific cerebral responses such as the amplitude of N170 or activation in face-selective occipitotemporal regions are reduced when faces are inverted than upright (Haxby et al., 1999; Rossion & Gauthier, 2002; Rossion et al., 2000; Yovel & Kanwisher, 2005). Overall, past studies indicated that the disruption of holistic processing via face inversion substantially impacts the way they are qualitatively perceived and represented at the cerebral level (Rossion, 2008). However, holistic processing of faces has been mainly investigated using fine-grained tasks such as face identification. In the context of rapid face detection, a couple of studies suggested that holistic processing can happen at a glance, for face exposure durations as short as 50 ms (Richler, Mack, Gauthier, & Palmeri, 2009; Taubert, Apthorp, Aagten-Murphy, & Alais, 2011). A study using a saccadic choice task in which stimuli were intact or filtered to preserve low or high spatial frequencies (Guyader et al., 2017) revealed faster saccadic reaction times (SRTs) toward faces than other target categories in all viewing conditions, but SRTs toward face targets were faster when the images were unfiltered and filtered in low spatial frequencies than when they were filtered in high spatial frequencies. These results suggested that rapid orienting responses toward faces could be mediated by fast processing of their low spatial frequency content, conveying coarse information, such as the global shape of faces and the configuration of their main features (i.e., the eyes, nose, and mouth). These results are supported by other findings showing that low spatial frequencies are predominantly extracted at early stages of face processing (Goffaux et al., 2010; Petras, Jacobs, & Goffaux, 2019). Critically, low spatial frequencies have also been shown to support holistic processing (Goffaux, Hault, Michel, Vuong, & Rossion, 2005; Goffaux & Rossion, 2006), leading to the hypothesis that ultra-rapid orienting responses toward faces filtered in low spatial frequencies could be mediated by their holistic processing. On the other hand, other studies suggested that the rapid orienting responses toward faces would rather be supported by the processing of isolated and salient facial features such as the eyes. For example, Lewis and Edmonds (2003) showed that face detection was strongly impaired when the eye area was masked, while masking other face parts such as the mouth had little effect.

The present study aimed to examine the extent to which (a) the ultra-rapid and (b) involuntary orienting responses toward faces previously observed using a saccadic choice task could rely on their holistic processing or on the processing of salient and isolated face features. In other words, are faces so rapidly and automatically detected because they are perceived holistically or because they contain features that our visual system is wired to rapidly detect? In order to address this question, we performed three experiments using a saccadic choice task adapted from previous studies (Crouzet et al., 2010; Guyader et al., 2017; Kauffmann et al., 2019; Kirchner & Thorpe, 2006). Participants were simultaneously presented with two images (e.g., one containing a face and the other one a vehicle) and had to initiate a saccade toward the one containing the target stimulus (e.g., the face), the other image being a distractor (e.g., the vehicle). In Experiment 1, the target and distractor images could be of two categories (face and vehicles), and we manipulated the orientation of stimuli, which were presented upright or inverted, the latter condition being used to disrupt holistic processing of faces. In Experiment 2, we further altered holistic processing of stimuli by dividing them into 2, 9, or 16 thumbnails that were randomly relocated (i.e., scrambled stimuli). We expected to replicate previous findings of faster and more involuntary saccadic responses toward faces than vehicles when stimuli were intact (i.e., shorter saccadic reaction times toward face than vehicle targets and more errors saccades toward face than vehicle distractors in the Upright condition of Experiment 1). Furthermore, if the rapid and involuntary orienting responses toward faces are mediated by their holistic processing, we expected these effects to be reduced when holistic processing was disrupted via stimulus inversion (Experiment 1) or scrambling (Experiment 2). Finally, Experiment 3 aimed at refining results of Experiment 2 to address the role of attributes such as color or shape and of the distractor category in driving the bias for faces.

## Experiment 1

### Material and method

#### Participants

Twenty-four participants (17 females; mean age ± *SD*: 22 ± 3 years) recruited from University of Grenoble Alpes with normal or corrected-to-normal vision were included in the experiment. All participants gave their informed written consent before taking part in the study, which was carried out in accordance with the Code of Ethics of the World Medical Association (Declaration of Helsinki) for experiments involving humans and was approved by the ethic committee of University of Grenoble Alpes (IRB00010290-2017-10-03-24). They received course credits for their participation.

#### Stimuli

**Figure 1.**
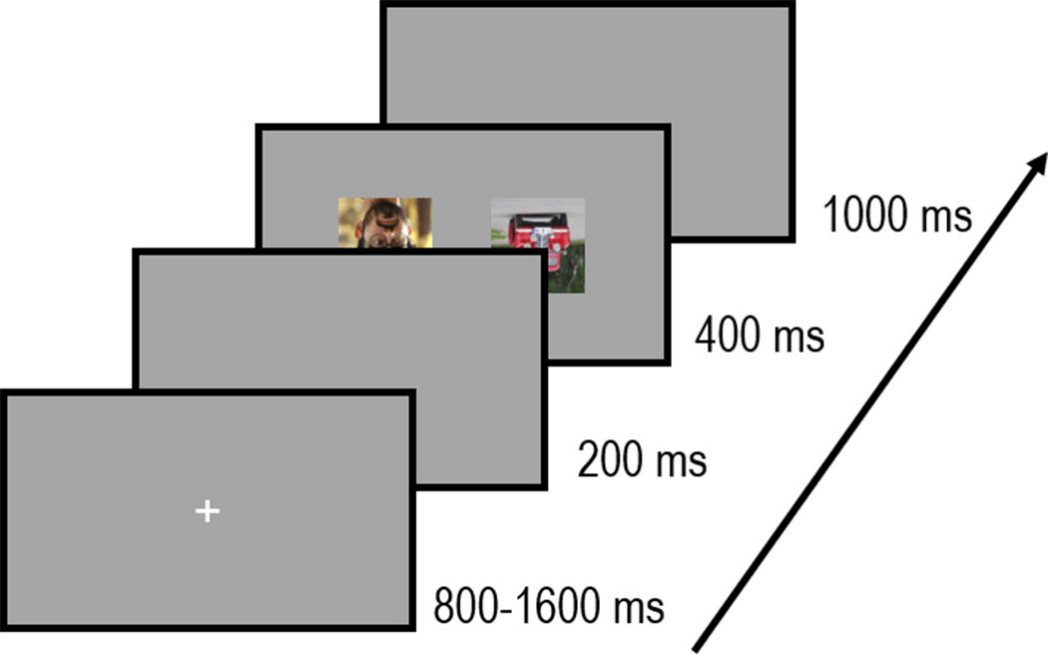
Time course of a trial in the Inverted condition. Participants had to fixate on a central cross displayed for 800–1,600 ms. After a gap (200 ms), two images simultaneously appeared for 400 ms, and the center of each image was lateralized at 8° of eccentricity. Participants were asked to initiate a saccadic eye movement as fast and as accurately as possible toward the image containing the target stimuli (a face or a vehicle).

Stimuli were created from 60 colored images downloaded from the free-from copyright “Pixabay” stock image base (https://pixabay.com/) under CC0 License.^1^ Half of the images contained human faces while the other half contained vehicles (e.g., cars, trucks, motorcycles; see Figure 1a). Original images were cropped to a square format (1,000 × 1,000 pixels) and resized to 300 × 300 pixels, subtending 11.5 × 11.5° of visual angle at a viewing distance of 58 cm. Cropping of stimuli was carefully done so that the main objects in the images (i.e., the face or the vehicle) had on average the same spatial location and size in both image categories. We subsequently confirmed this by manually delineating the main object in each image using a rectangle box and extracting its center (cf. Table 1). As indicated in Table 1, center of the face and vehicle in stimuli corresponded on average to the center of the image. Two versions of each image were built to create two Orientation conditions: one version in which the image was displayed it its canonical orientation (“Upright” condition) and the other one in which images were flipped upside-down (“Inverted” condition; cf. Figure 1). Mean luminance and root mean squared (RMS) contrast of each image were equalized to match the mean luminance (0.44 for luminance values between 0 and 1) and RMS contrast (0.25) of the whole set of images. Stimuli can be downloaded from https://osf.io/sahkf/.

**Table 1.** Mean center ± *SD* and size on the horizontal (X) and vertical (Y) axes of the main object (face or vehicle) in the images used in Experiment 1 (image size 300 × 300 pixels, X_0_ and Y_0_ being the top-left corner).

Characteristic	Faces, pixels	Vehicles, pixels	Two-sample *t* test
Mean center ± *SD* in X	151 ± 8	152 ± 10	*t*_29_ = 0.36, *p* = 0.72
Mean center ± *SD* in Y	150 ± 7	152 ± 9	*t*_29_ *=* 1.07, *p* = 0.29
Mean width ± *SD*	212 ± 37	221 ± 33	*t*_29_ = 0.89, *p* = 0.38
Mean height ± *SD*	247 ± 34	230 ± 35	*t*_29_ = 1.67, *p* = 0.11

#### Procedure

Stimuli were displayed using the Psychtoolbox (Brainard, 1997; Pelli, 1997) implemented in MATLAB R2016a (MathWorks, Natick, MA, USA) against a gray background (luminance of 0.44) on a 23.6-in. LCD monitor with a spatial resolution of 1,360 × 768 pixels, a refresh rate of 60 Hz, and a mean gray luminance of 51 cd/m^2^. Participants were seated at a distance of 58 cm from the screen. Their head was maintained by a chin- and forehead rest. Eye movements were recorded using an Eyelink 1000 eye-tracker (SR Research) with a sampling rate of 1,000 Hz and a nominal spatial resolution of 0.01° of visual angle. Only the right eye was recorded in each participant using the “pupil-corneal reflection” mode. The Eyelink software automatically detected saccades with the following thresholds: speed >30°/s, acceleration >8,000°/s2, and saccadic displacement >0.15°. Fixations were detected when the pupil was visible, and no saccade was in progress. Blinks were detected during partial or total occlusion of the pupil. Each session was preceded by a calibration procedure during which participants had to orient their gaze toward nine separate dots appearing sequentially in a 3 × 3 grid that occupied the entire display. A drift correction was performed every 10 trials. A new calibration was done in the middle of the experiment and when the drift error was above 0.5°.

All participants underwent two experimental sessions during the experiment, one for which the targets were images containing human faces (and the distractors were vehicle images) and the other one for which the targets were images containing vehicles (the distractors were human face images). The order of sessions was counterbalanced between participants. The procedure and task were exactly the same as in previous studies using a saccadic choice task (Crouzet et al., 2010; Guyader et al., 2017; Kauffmann et al., 2019). For each session, a trial started with a white fixation cross subtending 0.73° of visual angle, displayed centrally for 800 to 1,600 ms (duration sampled from a uniform distribution) and followed by a gap (mean gray-level screen) of 200 ms. Following the gap, two images (a target and a distractor) appeared simultaneously on the left and the right of the display for 400 ms. The center of each image was lateralized at 8° from the center of the screen. The intertrial interval was fixed at 1,000 ms (see Figure 1b). Participants were instructed to make a saccade as fast as possible toward the target image. In half of the trials, images were displayed upright (“Upright” condition) while they were flipped upside-down in the other half (“Inverted” condition). The presentation order of Upright and Inverted images was randomized. Each upright image and inverted image was presented twice in each session, once in each visual field. In total, there were 120 trials in each session (30 pairs of vehicle and 30 pairs of face images × 2 orientation conditions × 2 sides of presentation). Before the experiment, participants completed a training session comprising eight trials in order to get familiarized with the task, using stimuli that were not subsequently used in the main experiment. The experiment lasted approximately 10 min in each session.

#### Data analysis

We analyzed the error rate (%) and latency of correct saccades (or correct SRT from stimulus onset, in milliseconds) of the first saccadic response following stimulus appearance. Trials in which (a) a blink occurred, (b) saccades were initiated within less than 50 ms after stimulus onset, (c) saccades were initiated from more than 2° around the fixation cross, and (d) saccades had an amplitude below 1° or (e) a duration above 100 ms were discarded from the analyses. This resulted in removing 10.2% of the trials.

For each parameter, the mean error rate (mER) and the mean latency (SRT), we performed repeated-measures analyses of variance (ANOVAs) with the Target category (face, vehicle), the Orientation condition (upright, inverted) and the target side (left, right) as within-subject factors. Analyses were performed using MATLAB R2016 (MathWorks) and Statistica 10 software (Statsoft, Tulsa, OK, USA). Effect sizes were estimated by calculating partial eta-squared (η_p_^2^). The significance level of tests was set at α = 0.05, and *p*-values corrected for the number of tests are reported for pairwise comparisons.

As done in previous studies using a saccadic choice task (Crouzet et al., 2010; Guyader et al., 2017; Kirchner & Thorpe, 2006), we also computed the minimum saccadic reaction times (minSRT) for each Target and Orientation condition. The minSRT corresponds to the minimum SRT for which there were significantly more correct than error saccades and was obtained as detailed below. The distributions of SRT were computed separately for correct and error saccades, taking all saccades of all participants. SRTs were grouped into 10-ms bins (e.g., the 120-ms bin contained SRT comprising between 115 and 124 ms). For each bin, the proportion of correct saccades was compared to the one of error saccades using a χ² test. If there was significantly more correct than error saccades in five consecutive bins, the first bin was then defined as the minSRT (for a similar procedure, see Crouzet et al., 2010; Guyader et al., 2017).

### Results

#### Error rate and SRT

**Figure 2.**
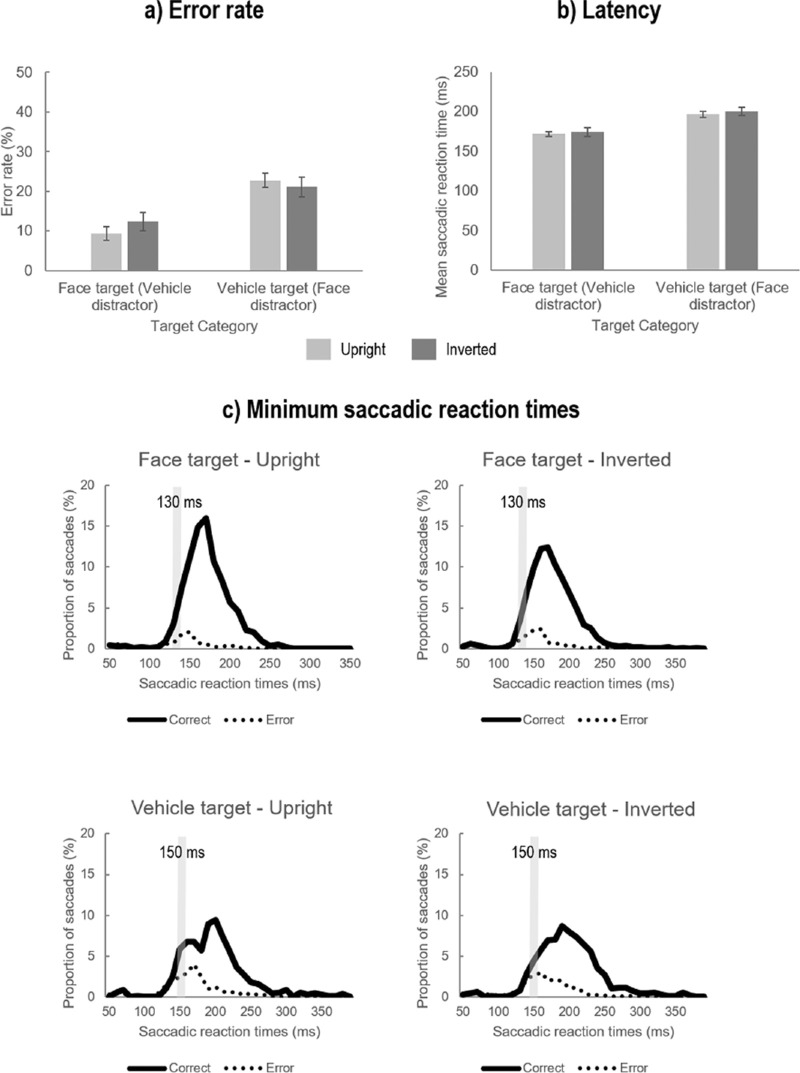
(a) Mean error rate (in % errors) and (b) mean latency or saccadic reaction time (in milliseconds) of saccades according to the Target category (Face, Vehicle) and the Orientation condition (Upright in light gray or Inverted in dark gray). Error bars correspond to ± 1 *SE*. (c) Distribution of saccadic reaction times for each Target category and each Orientation condition. Green lines correspond to correct saccadic responses and red lines to erroneous saccadic responses. The gray bars indicate the minimum 10-ms bin at which the proportion of correct saccades was significantly higher than error saccades in five consecutive bins.

The ANOVA performed on mean error rates (mER; cf. Figure 2a) revealed a main effect of target category (*F*_1, 23_ = 39.05, *p* < 0.0001, η_p_^2^ = .629). Participants made more errors when the target was a vehicle (i.e., the distractor was a face, mean ± *SD*: 21.84 ± 10.80%) than when it was a face (i.e., the distractor was a vehicle; 10.87 ± 8.16%). There was no main effect of the Target side (*F*_1, 23_ = 3.20, *p* = 0.09, η_p_^2^ = .122) or the Orientation condition (*F*_1, 23_ = 0.45, *p* = 0.51, η_p_^2^ = .019). However, the Orientation condition significantly interacted with the Target category (*F*_1, 23_ = 6.66, *p* = 0.02, η_p_^2^ = .225), suggesting that the difference in mER between face and vehicle targets was reduced in the Inverted relative to the Upright condition. Further pairwise comparisons revealed that participants made more errors when the target was a vehicle than when it was a face in both Orientation conditions (Face target—Upright: 9.37 ± 8.22%, Vehicle target—Upright: 22.75 ± 11.23%, *p* < 0.0001; Face target—Inverted: 12.37 ± 8.81%, Vehicle target—Inverted: 21.12 ± 12.03%, *p* < 0.0005). However, when the target was a face, they made more errors in the Inverted than Upright condition (*p* = 0.004) while there was no significant difference in mER according to the Orientation condition when the target was a vehicle (*p* > 0.99) suggesting that the proportion of error saccades toward face distractors was not influenced by the Orientation condition. All other interactions were not significant (Target category × Target side; Target side × Orientation condition; Target category × Orientation condition × Target side; all *F*_1, 23_ < 1).

The analysis of mean SRT (cf. Figure 2b) revealed a main effect of the Target category (*F*_1, 23_ = 40.78, *p* < 0.0001, η_p_^2^ = .639) and a main effect of the Orientation condition (*F*_1, 23_ = 10.43, *p* = 0.004, η_p_^2^ = .312). Participants initiated their saccadic response toward the target faster when it was a face (173 ± 15 ms) than a vehicle (198 ± 27 ms). Furthermore, they were also slightly faster to initiate saccadic responses when stimuli were upright (184 ± 19 ms) than inverted (187 ± 20 ms), although this difference was only of 3 ms. There was no main effect of the Target side (*F*_1, 23_ < 1) and no significant interaction between any of the manipulated factors (Target category × Orientation condition: *F*_1, 23_ = 0.24, *p* = 0.63; Target category × Target side: *F*_1__, 23_ = 1.83, *p* = 0.19; Target side × Orientation condition: *F*_1__, 23_ < 1; Target category × Orientation condition × Target side: *F*_1__, 23_ = 1.11, *p* = 0.30).

#### Minimum saccadic reaction time

The analysis of minSRT (cf. Figure 2c) revealed that when stimuli were upright, the fastest saccades correctly initiated toward face targets corresponded to the 130-ms bin (i.e., SRT comprised between 125 and 134 ms), while more time was needed to initiate correct saccades toward vehicle targets for which the minSRT corresponded to the 150-ms bin (i.e., SRT comprised between 145 and 154 ms). Critically, minSRT were exactly the same when stimuli were inverted: The minSRT toward inverted face targets corresponded to the 130-ms bin and the minSRT toward inverted vehicle targets corresponded to the 150-ms bin.

#### Accuracy profiles as a function of saccadic reaction time

The analysis of error rates revealed that participants made more errors when they had to perform saccades toward inverted than upright face targets, suggesting that disruption of holistic processing overall impaired the ability to detect faces. However, given that the mean latency and minSRT of correct saccades toward face targets did not differ between the Inverted and Upright conditions, it is unclear whether the higher error rate for inverted faces concerned early face detection associated with ultra-rapid saccades or later stages of this early processing. In order to address this question, we used an approach similar to the analysis of minimum saccadic reaction times: For each saccadic response time bin, we computed and compared the proportion of error saccades in the Upright and Inverted Face target conditions using χ² tests. Results revealed that the proportion of error saccades in the Inverted and Upright face target conditions only started to differ for saccades with saccadic reaction times corresponding to the 160-ms time bin (i.e., saccadic reaction times between 155 and 164 ms). Importantly, this suggests that the higher error rate of saccades toward inverted than upright face targets occurred for saccades with latencies well above the minimum saccadic reaction time (i.e., 125- to 134-ms time bin) and therefore did not concern the earliest saccadic responses toward face targets. In other words, these results indicate that the ultra-rapid saccades toward inverted or scrambled face targets were not associated with a higher error rate than saccades toward intact faces.

In summary, these analyses indicate that, although participants made more errors to saccade toward face targets when they were inverted than upright, the speed of saccadic responses (as assessed by averaged and minSRTs) as well as the proportion of involuntary orienting responses toward face distractors (higher rate of error saccades toward face than vehicle distractors) were not affected by stimulus inversion. This therefore suggests that ultra-rapid and involuntary saccades toward face stimuli cannot be accounted for by their holistic processing, which was disrupted in the Inverted condition. It is thus likely that the bias for faces relies on other processes, such as the fast detection of specific face features, processed independently from each other. In order to further address this, we subsequently examined the ending points of saccades within face and vehicle images. We expected that if participants’ gaze was mainly attracted by a specific feature in face targets, in both Orientation conditions, we should observe a difference between the ending location of saccades on the vertical axis (Y) according to the Orientation condition when the target is a face but not when is it a vehicle. However, saccadic endpoints on the horizontal axis (X) should not be influenced by the Orientation condition.

#### Saccadic endpoints

For these analyses, the coordinates of the endpoint of correct saccades in X and Y (in degrees) within the display were extracted and brought back into image space (i.e., within a square of 11.5 × 11.5°, X_0_ and Y_0_ coordinates being the center of the image). This allowed comparisons between saccadic endpoints within the images, irrespective of their side (left or right) on the display. Positive (negative) X and Y coordinates correspond to rightward (leftward) and upper (lower) locations relative to the image center, respectively. We then performed 2 × 2 × 2 ANOVAs on mean X and Y endpoints with the Target category, the Orientation condition, and the Target side as within-subject factors.

The ANOVA performed on mean saccadic endpoints in X revealed no main effect of Target category (*F*_1, 23_ < 1) or Orientation condition (*F*_1, 23_ < 1) or interaction between these two factors (*F*_1, 23_ = 1.33, *p* = 0.26). However, there was a main effect of Target side (*F*_1, 23_ = 63.54, *p* < 0.0001, η_p_^2^ = .734), suggesting that saccades landed on the right half of the image when stimuli were on the left visual field (1.07 ± 0.61°) while the opposite was found for stimuli displayed in the right visual field (−0.71 ± 0.66°). This effect indicates that participants generally performed their saccades toward the half part of the image that was the closest from the central fixation point. Furthermore, the Target side significantly interacted with the Target category (*F*_1, 23_ = 41.18, *p* < 0.0001, η_p_^2^ = .646). Pairwise comparisons revealed that when stimuli were displayed on the left visual field, saccades toward vehicle targets (1.42 ± 0.86° pixels) landed more rightward in the images (i.e., closer to the central fixation point) than saccades toward face targets that landed closer to the image center (0.72 ± 0.54°, *p* = 0.0005). Similarly, when stimuli were displayed on the right visual field, saccades toward vehicle targets (−1.02 ± 0.72°) landed more leftward in the images (i.e., closer to the central fixation point) than saccades toward face targets (−0.40 ± 0.66°, *p* < 0.0001). This result is consistent with previous findings of larger saccades toward face than vehicle targets (Kauffmann et al., 2019).

The ANOVA performed on saccadic endpoints in Y revealed no main effect of the Target category (*F*_1, 23_ = 2.34, *p* = 0.14), but there was a main effect of the Orientation condition (*F*_1, 23_ = 61.49, *p* < 0.0001, η_p_^2^ = .728). Participants’ gaze was oriented more upward in the Inverted (0.12 ± 0.43°) than in the Upright condition (−0.15 ± 0.36°). Importantly, there was a significant interaction between these two factors (*F*_1, 23_ = 101.44, *p* < 0.0001, η_p_^2^ = .815). In line with our assumptions, pairwise comparisons revealed that when the target was a face, participants oriented their gaze in opposite sides around the vertical axis relative to the image center when stimuli were upright (−0.34 ± 0.29°) than inverted (0.22 ± 0.47°, *p* < 0.0001). However, there was no significant difference between Y saccadic endpoints when the target was a vehicle (upright: 0.04 ± 0.40°; inverted: 0.02 ± 0.44°, *p* = 0.99). We also found a main effect of the Target side (*F*_1, 23_ =12.37, *p* = 0.002, η_p_^2^ = .350), indicating that participants’ gaze was oriented slightly more upward when stimuli were displayed in the left (0.09 ± 0.37°) than in the right (−0.12 ± 0.45°) visual field. However, this factor did not significantly interact with the other factors of interest (all *F*s < 1).

**Figure 3.**
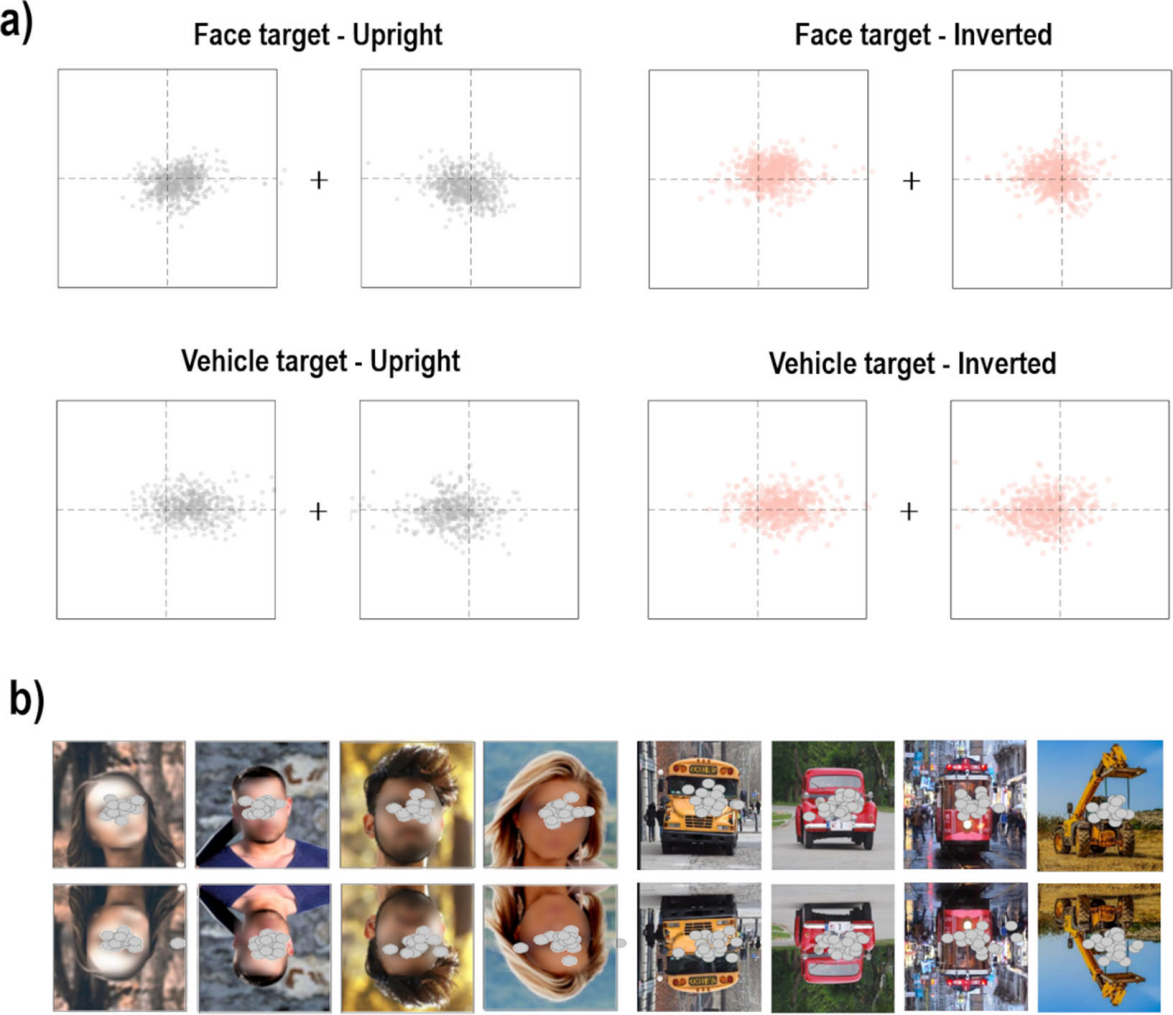
(a) Endpoints of correct saccades of all participants and all trials for each Target category (Face and Vehicle) and Orientation condition (Upright and Inverted). The squares correspond to the border of left and right lateral images on the display, relative to the center of the screen, represented by the cross. The dotted lines indicate the vertical and horizontal midlines of images. (b) Examples of individual saccadic endpoints plotted on Upright (top row) and Inverted (bottom row) face (left) and vehicle (right) stimuli. The images used in the experiment and in the figure are under the Pixabay license (https://pixabay.com), which allows free-from-copyright use and modification of images. It should be noted that faces were blurred for publication purposes only.

**Figure 4.**
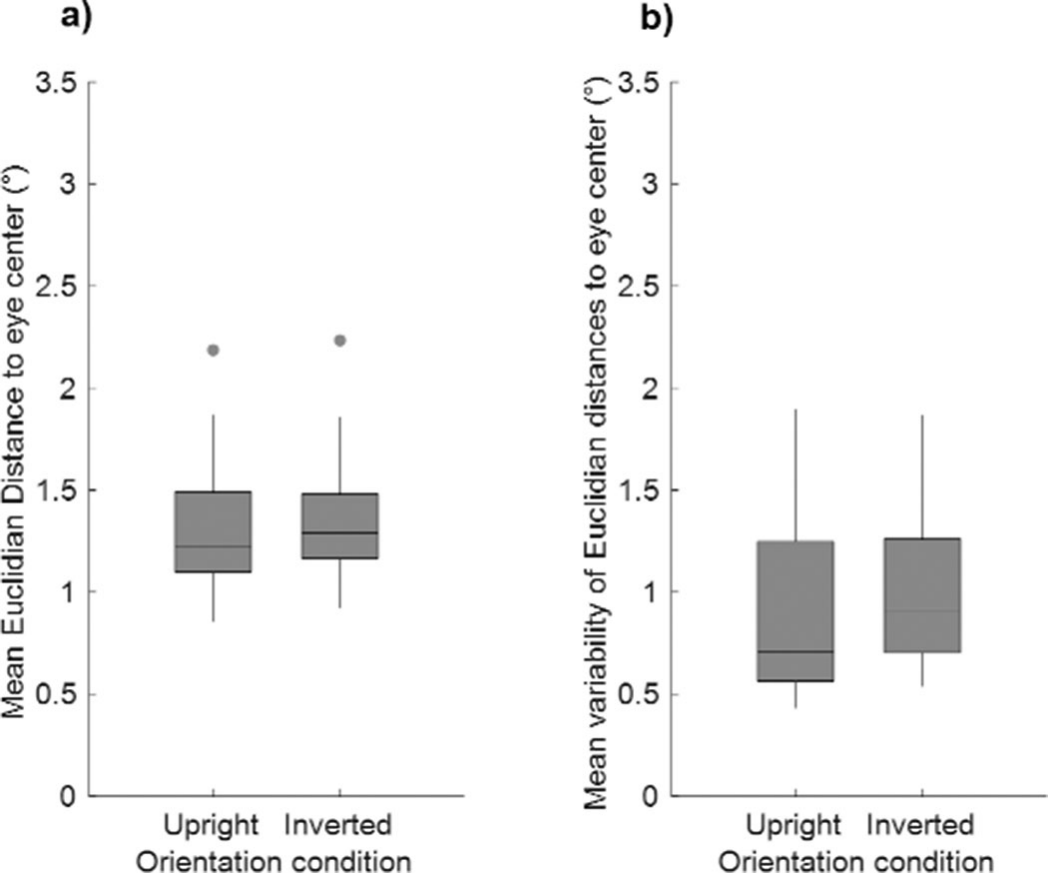
Boxplots of (a) mean Euclidian distances (in degrees) of saccade endpoints to the eye center in images and (b) mean between-subject variability of these distances (in degrees) according to the Orientation conditions (Upright and Inverted). The lower and upper limits of the box correspond to the first and third quartiles, respectively, and the lower and upper limits of the vertical bars to the first and ninth deciles. The horizontal bar indicates the median, and the dots correspond to extreme values.

Results of these analyses therefore support the idea that when the target was a face, participants oriented their gaze toward the same feature that was vertically flipped according to the Orientation condition, while there was no evidence for such pattern when the target was a vehicle. It should be noted that because our stimuli were built from natural images, they were not standardized in terms of size or spatial location of face features, which could be very variable between images. Therefore, the nature of the targeted feature in face stimuli could not be inferred from mean saccadic endpoints. In order to address this question, we plotted individual endpoints of saccades on each image (see examples in Figure 3). Visual inspection of these plots revealed that almost all participants’ saccades landed around the eye region for face targets, whether they were upright or inverted. In order to quantitatively assess this, we delineated the eye region in all face images using a rectangle box centered between the two eyes (encompassing the eyebrows) and computed their center and size in image space (mean ± *SD* X_centre_: 0.04 ± 0.73°; Y_centre_: −0.08 ± 0.5°; X_size_: 4.31 ± 0.70 °; Y_size_: 1.44 ± 0.41°). It should be noted that in the majority of face images, the eyes were located in the inferior part of the images in the Upright condition and therefore in the superior part of the image in the Inverted condition. We then calculated for each image the mean Euclidian distance between the landing position of participants’ saccadic responses (i.e., the endpoint of correct saccades) and the center of the eye region and found that on average, saccadic endpoints were located within a distance below 1.5° from the eye center in both Orientation conditions (Upright: mean distance ± *SD*: 1.29 ± 0.30° within the range [0.86–2.18°]; Inverted: mean distance ± *SD*: 1.35 ± 0.29° within the range [0.92–2.23°]; see Figure 4a) with a mean between-subject variability averaged across images (i.e., mean of *SD*) of about 1° (Upright: mean *SD* of distance ± *SD*: 0.90 ± 0.42° within the range [0.43–1.90°]; Inverted: mean *SD* of distance ± *SD*: 1.02 ± 0.39° within the range [0.54–1.87°]; see Figure 4). Paired *t* tests on mean distances and mean between-subjects variability of distances per face images did not show a significant difference between the two Orientation conditions (mean distances: *t*_29_ = −1.20, *p* = 0.48; mean between-subject variability of distances: *t*_29_ = −1.14, *p* = 0.52).

Finally, we tested the Pearson correlation between mean saccadic endpoints per face image and the center of its eye region in X and Y. We found significant positive correlations for each Orientation condition, indicating that the higher (lower) the center of the eye region in face images, the higher (lower) the mean saccadic endpoints in Y (Upright: *r* = .89, *p* < 0.0001; Inverted: *r* = .80, *p* < 0.0001), and the more rightward (leftward) the center of the eye region in face images, the more rightward (leftward) the mean saccadic endpoints in X (Upright: *r* = .69, *p* < 0.0001; Inverted: *r* = .68, *p* < 0.0001). These results therefore further support the idea that participants gaze consistently targeted the eyes in face images.

### Discussion of experiment 1

Results of Experiment 1 replicated previous findings (Boucart et al., 2016; Crouzet et al., 2010; Crouzet & Thorpe, 2011; Guyader et al., 2017; Kauffmann et al., 2019) by showing that upright face stimuli elicit (a) more involuntary saccades (as reflected by the higher rate of error saccades toward face than vehicle distractors) and (b) faster saccadic responses toward them than do vehicle stimuli during a saccadic choice task. Furthermore, the minimum saccadic reaction time observed for face targets was similar to what was reported by previous studies (i.e., ∼130 ms; Crouzet et al., 2010; Guyader et al., 2017). In the present experiment, we manipulated the orientation of stimuli that were presented upright or inverted, the latter condition being used to disrupt holistic processing of faces. Analysis revealed that participants tended to make more errors when stimuli were inverted than upright in the face Target condition. However, for correct saccades, the speed of saccadic responses toward faces was not affected by stimulus inversion. The discrepancy between results in terms of accuracy and saccadic reaction times may be explained by the fact that while the accuracy measure is based on the proportion of errors, saccadic reaction times only took into account correct saccades. In that context, our results indicate that disruption of holistic processing via face inversion impaired the ease or precision of face detection, leading to overall more errors. Furthermore, the analysis of error profiles as a function of saccadic reaction times suggested that ultra-rapid saccades toward faces targets (with a minSRT of 130 ms) were not associated with a higher error rate in the Inverted relative to the Upright condition and that impairment of face detection due to inversion was rather observed for relatively longer saccadic reaction times. Analyses of saccadic endpoints in this experiment further revealed that participants consistently oriented their saccades toward the eye region in faces, in both Orientation conditions. Overall, these results suggest that ultra-rapid and involuntary orienting responses toward faces cannot be explained by their holistic processing and may rather be supported by the fast detection of salient face features processed in isolation such as the eyes. These latter results would be in agreement with previous studies highlighting the role of the eyes for rapid face detection, by showing, for example, that masking the eye part of faces impairs their detection (Lewis & Edmonds, 2003).

**Figure 5.**
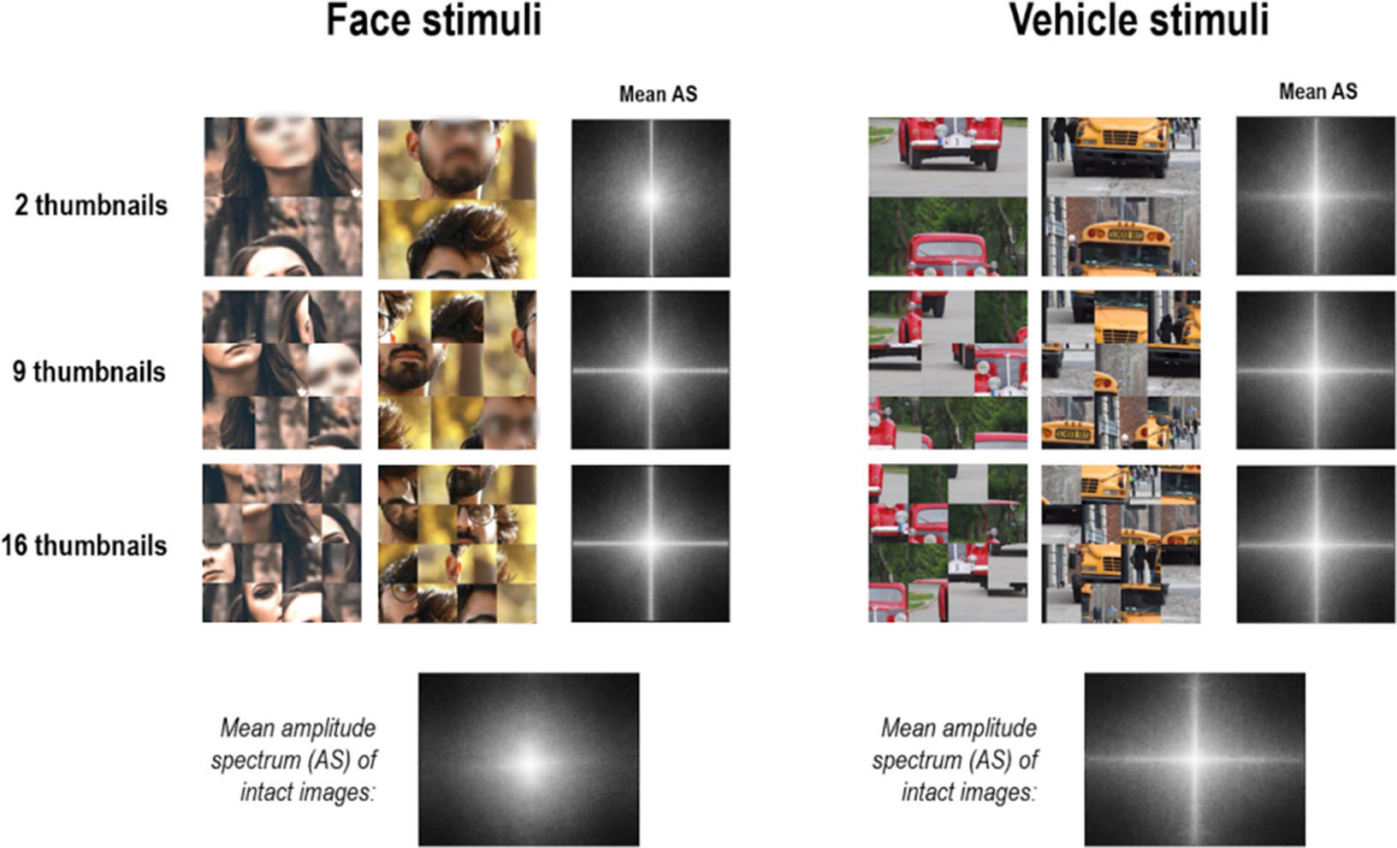
Examples of scrambled stimuli used in Experiment 2. It should be noted that faces were blurred for publication purposes only. Images were divided into 2, 9, or 16 thumbnails, which were randomly relocated. The mean amplitude spectra (AS) of each Scramble condition are shown on the right part of each panel, and mean amplitude spectra of intact face and vehicle images are displayed below for comparison. The images used in the experiment and in the figure are under the Pixabay license (https://pixabay.com), which allows free-from-copyright use and modification of images.

However, there are alternative explanations for these results. First, it has been shown that eye movements toward an object tend to land around its center of gravity (Findlay, 1982), which usually corresponds to the eye region in faces (Bindemann, Scheepers, & Burton, 2009). It is thus possible that saccades toward the eyes observed in our experiment actually result from this center-of-gravity bias rather than from the detection of the eyes per se. It should also be noted that while face inversion in this experiment disrupted the spatial configuration of facial features, it did not alter lower-level properties of images such as their amplitude spectra, which substantially differs between face and vehicle stimuli (e.g., Guyader et al., 2017—see Figure 5). It is thus possible that such low-level differences also play a role in the fast orienting bias toward faces. Indeed, previous studies have shown that the visual system relies on the rapid extraction of these low-level features for rapid face detection (Crouzet & Thorpe, 2011; Guyader et al., 2017; Honey, Kirchner, & VanRullen, 2008; Vanrullen, 2006).

In order to (a) examine the role of the eye region in driving ultra-rapid saccades toward faces, relative to a center-of-gravity bias due to the central location of the eyes within upright and inverted faces and (b) to better control for low-level differences between face and vehicle stimuli, we conducted a second experiment in which the same saccadic choice task was used, but this time, holistic processing of faces was disrupted by dividing our stimuli into thumbnails that were randomly relocated (i.e., scrambled stimuli). This scrambling procedure allowed us to disrupt holistic processing while preserving isolated face parts such as the eyes and ensure that the eye region would not necessarily fall at the center of images. It also allowed us to alter low-level properties of stimuli by introducing more energy in the horizontal and vertical orientations, making face and vehicle stimuli more similar in terms of their amplitude spectra (see Figure 5). We used different levels of scrambling (i.e., 2, 9, or 16 thumbnails) in order to assess various degrees of alteration of face parts configuration. If the ultra-rapid saccades toward faces are mediated by the detection of salient features such as the eyes, irrespective of their location, or by low-level properties of images, we expected that the scrambling of stimuli should have little effect on the latency and proportion of involuntary saccades toward face targets. Furthermore, we expected saccadic endpoints to be located near the thumbnail(s) containing the eye(s) in scrambled stimuli, irrespective of their spatial location relative to other face parts.

## Experiment 2

### Participants

Twenty-three participants (20 females; mean age ± *SD*: 21 ± 3 years) who did not take part in Experiment 1 and had normal or corrected-to-normal vision were recruited from University of Grenoble Alpes and included in the experiment. All participants gave their informed written consent before taking part in the study, which was carried out within the same ethical framework as in Experiment 1.

#### Stimuli

Stimuli were created from the same 60 images used in Experiment 1. In the present experiment, we altered the spatial configuration of stimuli by dividing the images into thumbnails that were randomly relocated (i.e., scrambled stimuli). For each image, we created three versions with different degrees of scrambling by increasing the number of thumbnails the images were divided into (2, 9, or 16 thumbnails of 300 × 150, 100 × 100, or 75 × 75 pixels; i.e., 11.5 × 5.8°, 3.85 × 3.85°, or 2.88 × 2.88°, respectively; see Figure 5). This scrambling procedure allowed us to progressively alter the spatial configuration and low-level properties of stimuli by increasing the number of thumbnails while preserving recognizable features (e.g., an eye for face stimuli or a headlamp for vehicle stimuli), allowing participants to perform the task.

#### Procedure

The procedure and task were the same as in Experiment 1. All participants underwent two experimental sessions during the experiment, one session for which the targets were human faces (and the distractors were vehicle images) and the other one for which the targets were images containing vehicles (the distractors were human face images). The order of sessions was counterbalanced between participants. All stimuli were presented twice (once on the right and once on the left) in each of their scrambling versions (2, 9, and 16 thumbnails) in a randomized order. In total, there were 180 trials in each session (30 pairs of stimuli × 3 scrambling conditions × 2 side of presentation). Before the experiment, participants completed a training session comprising 12 trials in order to get familiarized with the stimuli and the task, using stimuli that were not subsequently used in the experiment. The experiment lasted approximately 12 min in each session.

#### Data analysis

As for Experiment 1, we analyzed the error rate (% error) and SRT (in milliseconds) of the first saccadic response following stimulus appearance and meeting the same criteria as in Experiment 1. This resulted in removing 19% of the trials.

For each parameter, the mean error rate and the mean SRT, we performed repeated-measures ANOVAs with the Target category (face, vehicle) and the Scramble condition (2, 9, 16 thumbnails) as within-subject factors. It should be noted that since the Target side had no effect on error rate and SRT in Experiment 1 and was not a factor of interest, this factor was not included in the present analysis. Effect sizes were estimated by calculating partial eta-squared (η_p_^2^). The significance level of tests was set at α = 0.05, and corrected *p*-values are reported for pairwise comparisons.

We also computed the minSRT for each Target category and Scramble condition using the same procedure as in Experiment 1.

### Results

#### Error rate and latency of saccadic responses

**Figure 6.**
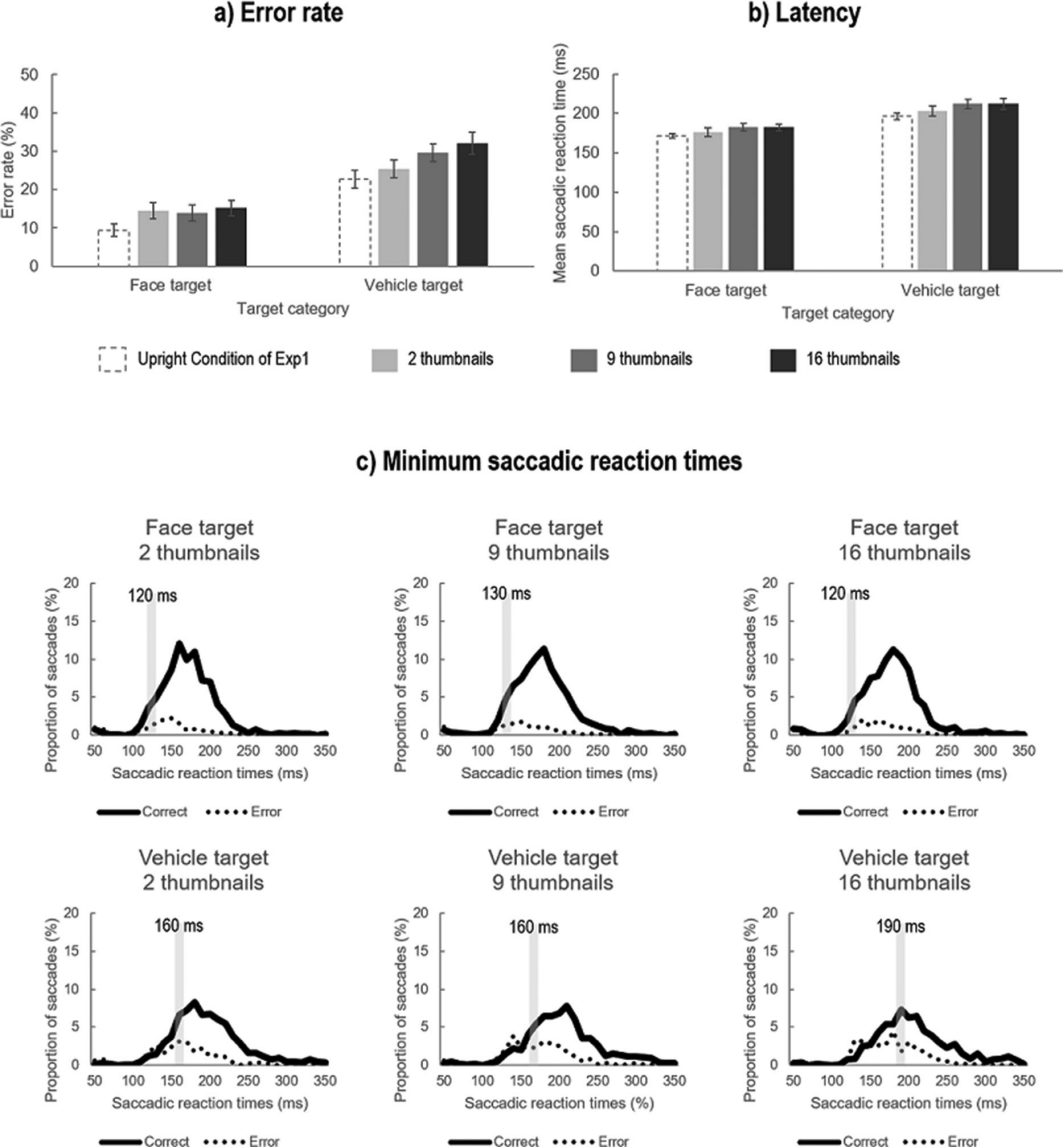
(a) Mean error rate (in % errors) and (b) latency or mean saccadic reaction times (in milliseconds) of saccades according to the Target category (Face, Vehicle) and the Scramble condition (2, 9, or 16 thumbnails). Results of the Upright condition of Experiment 1 (bars with dotted lines) are also shown for comparison. Error bars represent ± 1 *SE*. (c) Distribution of saccadic reaction times for each Target category and each Scramble condition. Green lines correspond to correct saccadic responses and red lines to erroneous saccadic responses. The gray bars indicate the minimum 10-ms bin at which the proportion of correct saccades was significantly higher than error saccades in five consecutive bins.

The ANOVA performed on mean error rates (mER; see Figure 6a) revealed a main effect of Target category (*F*_1, 22_ = 53.82, *p* < 0.0001, η_p_^2^ = .710) and a main effect of the Scrambling condition (*F*_1, 22_ = 4.12, *p* = 0.02, η_p_^2^ = .158). Participants made more error saccades when the target was a vehicle (i.e., the distractor was a face, mean ± *SD*: 29.00 ± 10.76%) than when it was a face (i.e., the distractor was a vehicle; 14.51 ± 8.76%). Furthermore, the error rate increased as the number of thumbnails in scrambled stimuli increased (2 thumbnails: 19.91 ± 8.85%; 9 thumbnails: 21.73 ± 9.25%; 16 thumbnails: 23.63 ± 9.81%). There was also a significant interaction between these two factors (*F*_1, 22_ = 3.44, *p* = 0.04, η_p_^2^ = .135). Further comparisons revealed that participants made more errors when the target was a vehicle than a face irrespective of the Scramble condition (Face target—2 thumbnails: 14.46 ± 10.31%, Vehicle target—2 thumbnails: 25.35 ± 10.79%, *p* = 0.001; Face target—9 thumbnails: 13.89 ± 10.25%, Vehicle target—9 thumbnails: 29.57 ± 11.16%, *p* < 0.0001; Face target—16 thumbnails: 15.17 ± 9.36%, Vehicle target—16 thumbnails: 32.09 ± 13.52%, *p* < 0.0001). Furthermore, polynomial contrasts for linear and quadratic trends (C_lin_ and C_quad,_ respectively) showed that when the target was a vehicle, the proportion of error saccades toward face distractors increased linearly with the number of thumbnails in scramble images (C_lin_: *p* = 0.006; C_quad_: *p* = 0.99) while there was no trend toward a linear increase of error rates toward vehicle distractors when the target was a face (C_lin_: *p* = 0.99; C_quad_: *p* = 0.99). Overall, these results suggest that while the alteration of spatial configuration increased the proportion of error saccades toward face distractors, it had little effect on the accuracy of saccades toward face targets.

The ANOVA performed on mean SRT (see Figure 6b) revealed a main effect of Target category (*F*_1, 22_ = 33.93, *p* < 0.0001, η_p_^2^ = .607) and a main effect of the Scramble condition (*F*_1, 22_ = 14.13, *p* < 0.0001, η_p_^2^ = .391). Participants initiated their saccadic response toward the target faster when it was a face (180 ± 20 ms) than a vehicle (209 ± 31 ms). Furthermore, they were also faster to initiate saccadic responses when stimuli were scrambled into 2 (190 ± 24 ms) than 9 (197.4 ± 24 ms) or 16 thumbnails (197 ± 24 ms). However, there was no significant interaction between these two factors (*F*_1, 22_ = 0.51, *p* = 0.61).

#### Minimum saccadic reaction times (minSRT)

In agreement with the analysis of mean saccadic reaction times, the analysis of minSRT (Figure 6c) revealed that the fastest correct saccades were initiated earlier when the target was a face than a vehicle. Importantly, the minSRT of saccades toward face targets was similar to the one observed for saccades toward intact face targets in Experiment 1 and remained relatively stable across each Scramble condition (2 thumbnails: 120-ms bin; 9 thumbnails: 130-ms bin; 16 thumbnails: 120-ms bin). In contrast, minSRT of saccades toward vehicle targets tended to increase with increasing alteration of stimuli spatial configuration (2 thumbnails: 160-ms bin; 9 thumbnails: 160-ms bin; 16 thumbnails: 190-ms bin).

In summary, these analyses indicate that the progressive alteration of stimuli's configuration and low-level properties according to the different Scramble conditions generally impacted performances by increasing reaction times and error rate of saccadic responses. However, these manipulations had little effect on the latency and accuracy of saccadic responses toward face targets, which were always faster and performed with less errors than saccades toward vehicle targets. Furthermore, the proportion of error saccades toward face distractors tended to increase with the scrambling level of stimuli. Based on Experiment 1, we expected that in the present experiment, the bias for face stimuli could have relied on the detection of the eyes in scrambled face images, irrespective of their location within the images or their relations with other face partis. In order to address this question, we performed an analysis on the distances of saccadic endpoints relative to the location of the eye(s) in scrambles stimuli as done for Experiment 1. For each image and each Scramble condition, we determined the location of the eye(s) and computed its/their center. We then calculated, for each stimulus, the mean Euclidian distances between the eye region and participants’ saccadic endpoint as well as their mean between-participant variability. When more than one thumbnail contained an eye in the scrambled stimulus, the distances were computed for each of these thumbnails, and only the shortest distance was taken into account.

**Figure 7.**
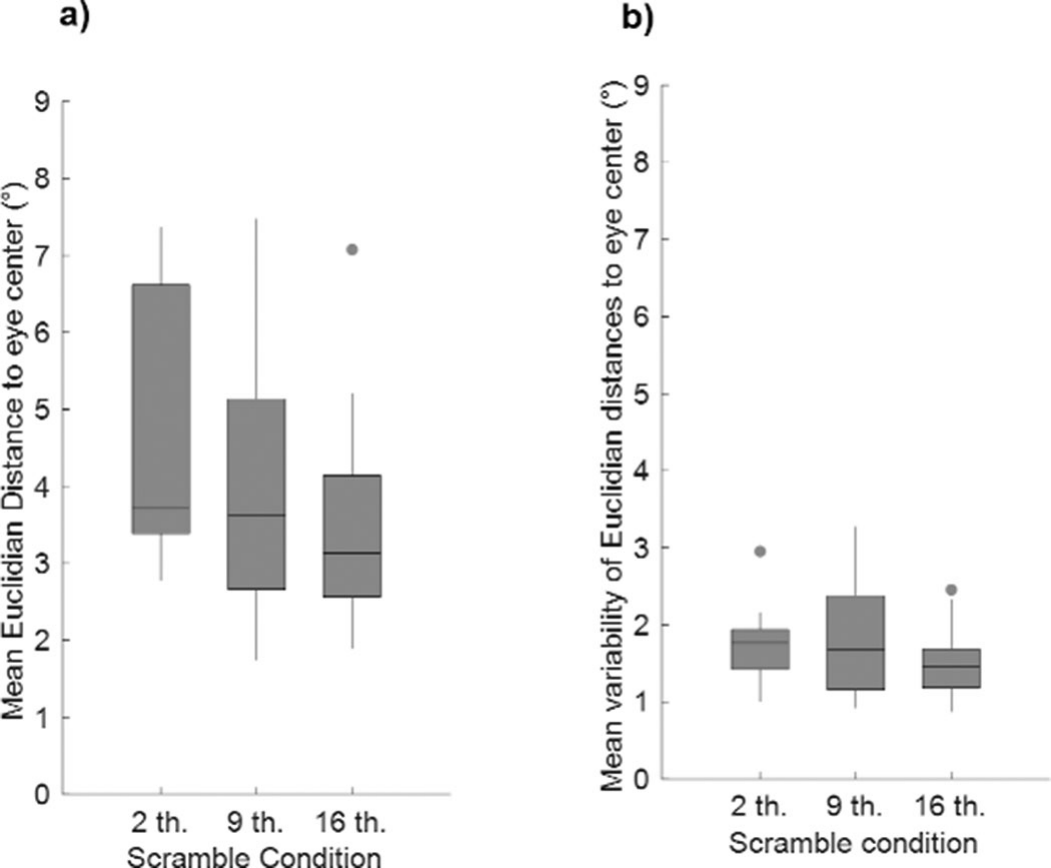
Boxplots of (a) mean Euclidian distances (in degrees) of endpoints of correct saccades to the eye center in images and (b) mean between-subject variability (i.e., mean of standard deviations) of these distances (in degrees) according to the Scramble condition. The lower and upper limits of the box correspond to the first and third quartiles, respectively, and the lower and upper limits of the vertical bars to the first and ninth deciles. The horizontal bar indicates the median, and the dots correspond to extreme values.

Contrary to what was expected, we found that in comparison to Experiment 1, saccadic responses landed further away from the eyes in stimuli, with mean distances above 3° (2 thumbnails: mean distance ± *SD*: 4.76 ± 1.69° within the range [2.78–7.36°]; 9 thumbnails: mean distance ± *SD*: 3.81 ± 1.53° within the range [1.74–7.48°]; 16 thumbnails: mean distance ± *SD*: 3.44 ± 1.13° within the range [1.90–7.08°]—see Figure 7a). Furthermore, the mean between-subject variability of these distances across stimuli was also higher than in Experiment 1 (2 thumbnails: 1.70 ± 0.40° within the range [1.01–2.95°]; 9 thumbnails: 1.81 ± 0.70° within the range [0.92– 3.27°]; 16 thumbnails: 1.52 ± 0.40° within the range [0.87–2.46°]—see Figure 7b). Paired *t* tests on mean distances and their mean between-subject variability per face stimuli revealed that mean distances to the eyes in face stimuli were lower for the 16-thumbnail than the 2-thumbnail Scramble condition while there was no significant difference between the 2- and 9-thumbnail or between the 9- and 16-thumbnail conditions (2 vs. 9 thumbnails: *t*_29_ = 2.13, *p* = 0.25, 2 vs. 16 thumbnails: *t*_29_ = −3.83, *p* = 0.004, 9 vs. 16 thumbnails: *t*_29_ = 1.13, *p* = 0.99). Furthermore, there was no difference between the three conditions in terms of mean between-subject variability of distances (2 vs. 9 thumbnails: *t*_29_ = −0.61, *p* = 0.99; 2 vs. 16 thumbnails: t_29_ = 1.81, *p* = 0.48; 9 vs. 16 thumbnails: *t*_29_ = 1.84, *p* = 0.46).

**Figure 8.**
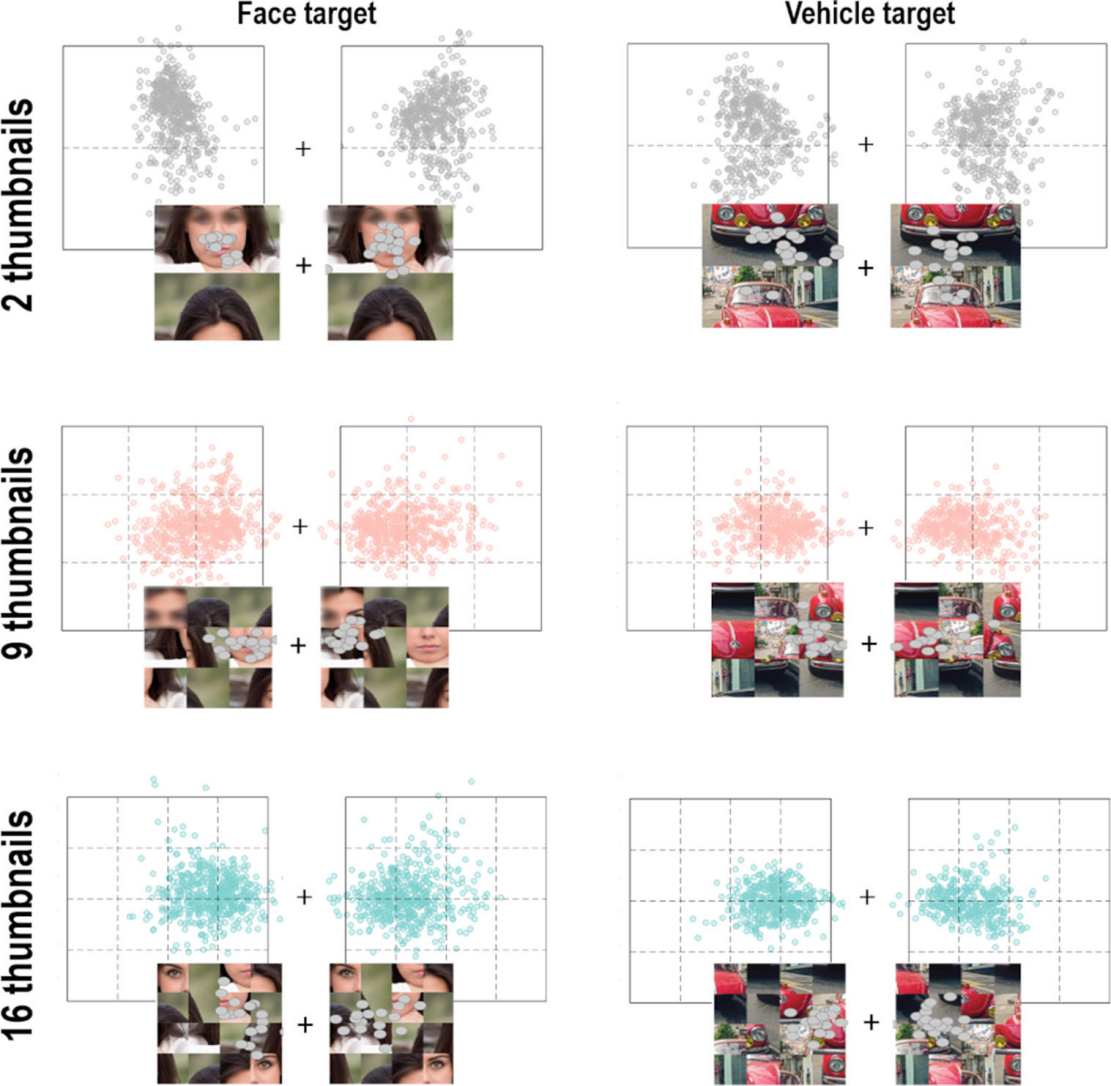
Impact points of correct saccades of all participants and all trials for each Target category and Scramble condition. The squares correspond to the border of left and right lateral images on the display, relative to the center of the screen, represented by the cross. The dotted lines indicate borders of thumbnails in each Scramble condition. Examples of individual saccadic endpoints plotted on stimuli appearing on the left or right side of the display are shown at the bottom. The images used in the experiment and in the figure are under the Pixabay license (https://pixabay.com), which allows free-from-copyright use and modification of images.

Overall, these results—confirmed by visual inspection of individual saccadic endpoints plotted on each stimulus (see examples in Figure 8)—suggest that contrary to what was observed in Experiment 1, participants’ gaze was not necessarily oriented toward the eyes in face stimuli when their holistic processing was altered with our scrambling procedure, even if some local configuration was preserved (as images in the 2- or 9-thumbnail condition). It actually appeared that no specific face feature was consistently targeted by participants, who rather tended to adopt a strategy to perform their saccades according to the Scramble condition, with saccades directed toward the upper part of the 2-thumbnail condition, and toward the thumbnails that were the closest from the central fixation point in the 9- and 12-thumbnail conditions, with similar patterns for both target categories (see Figure 8).

### Discussion of experiment 2

Results of Experiment 2 further supported the findings of Experiment 1 by showing that disruption of holistic processing via a scrambling procedure had no significant impact on the speed of orienting responses toward faces, as saccadic responses exhibited comparable minimum saccadic reaction times with those observed in the Intact condition of Experiment 1. Furthermore, these latencies remained comparable across the three Scrambling conditions, suggesting that they were not affected by the progressive alteration of face parts configuration. However, contrary to what was expected based on the analysis of saccadic endpoints in Experiment 1, participants did not necessarily target the eye region in scrambled faces in the present experiment. These results therefore suggest that irrespective of their content (e.g., an eye, a nose), isolated face parts may be sufficient to elicit rapid and involuntary eye movements toward faces.

However, it cannot be totally ruled out that other factors can account for the bias for faces observed in the present experiment. Indeed, it is possible that this bias simply relies on the processing of attributes such as their color, shape (e.g., curved lines), or higher-level attributes such as the degree of animacy that are characteristic of faces and strongly differ with the vehicle category for which these attributes were more variable. Indeed, although previous studies using a saccadic choice task have shown that the bias in favor of faces persists even when stimuli are gray-scaled (Boucart et al., 2016; Crouzet et al., 2010; Guyader et al., 2017) or when the target and distractors share more similarities in terms of animacy, shape, and degree of structural homogeneity (Boucart et al., 2016; Guyader et al., 2017; Nakano, Higashida, & Kitazawa, 2013), these studies used intact stimuli and it is thus possible that such attributes play a role when stimuli are scrambled as in our experiment.

Another intriguing finding of Experiment 2 was that while we expected that the proportion of error saccades toward face distractors would be reduced if their attentional capture was diminished by the disruption of holistic and configural processing, this proportion actually increased with the scrambling levels, suggesting that faces captured attention even more under these conditions. However, an alternative interpretation would be that the higher proportion of error saccades toward faces with the scrambling level does not reflect their higher attentional capture but rather the reduced attractiveness of vehicle targets. Unfortunately, the design of Experiment 2, in which only two categories of targets/distractors were used, does not allow us to disambiguate that question.

Experiment 3 was conducted in order to address (a) the role of attributes such as color or shape, as well as of higher-level attributes such as the degree of animacy of stimuli in driving ultra-rapid and involuntary orienting responses toward scrambled faces in Experiment 2, and (b) whether the higher proportion of error saccades toward face distractors actually reflect their higher attentional capture. In this experiment, we used intact and scrambled stimuli similar to the 16-thumbnail condition of Experiment 2 (in which the local configuration of faces was more strongly altered) but we added two additional target/distractor categories: flowers and animals. The flower category was chosen because it shares more similarities with the face category in terms of structural homogeneity characterized by a round shape. The animal category was chosen in order to match faces in terms of animacy. In order to address the role of color, we also manipulated the color content of stimuli that were presented in color or gray-scaled. Participants performed the same saccadic choice task as in Experiments 1 and 2 with the four target conditions (Faces, Vehicles, Animals, Flowers). For each target condition, the distractor could be of the three remaining categories. This additionally allowed us to examine the proportion of error saccades as a function of the distractor category. We expected that if the bias for face observed is primarily based on the processing of attributes such as color, this bias should be reduced or even disappear when stimuli are gray-scaled. Furthermore, if this bias is mainly based on the detection of attributes such as (a) their round shapes or (b) their degree of animacy, such a bias should be also observed for (a) the flower or (b) animal categories, respectively. Finally, if the higher proportion of error saccades toward face distractors observed in Experiment 2 does reflect their higher attentional capture, we expected participants to make more error saccades toward face than animal, flower, or vehicle distractors.

## Experiment 3

### Material and method

#### Participants

Sixteen participants (2 males; mean age ± *SD*: 27 ± 8 years) recruited from University of Grenoble Alpes with normal or corrected-to-normal vision were included in the experiment. All participants gave their informed written consent before taking part in the study, which was carried out within the same ethical framework as Experiments 1 and 2.

#### Stimuli

**Figure 9.**
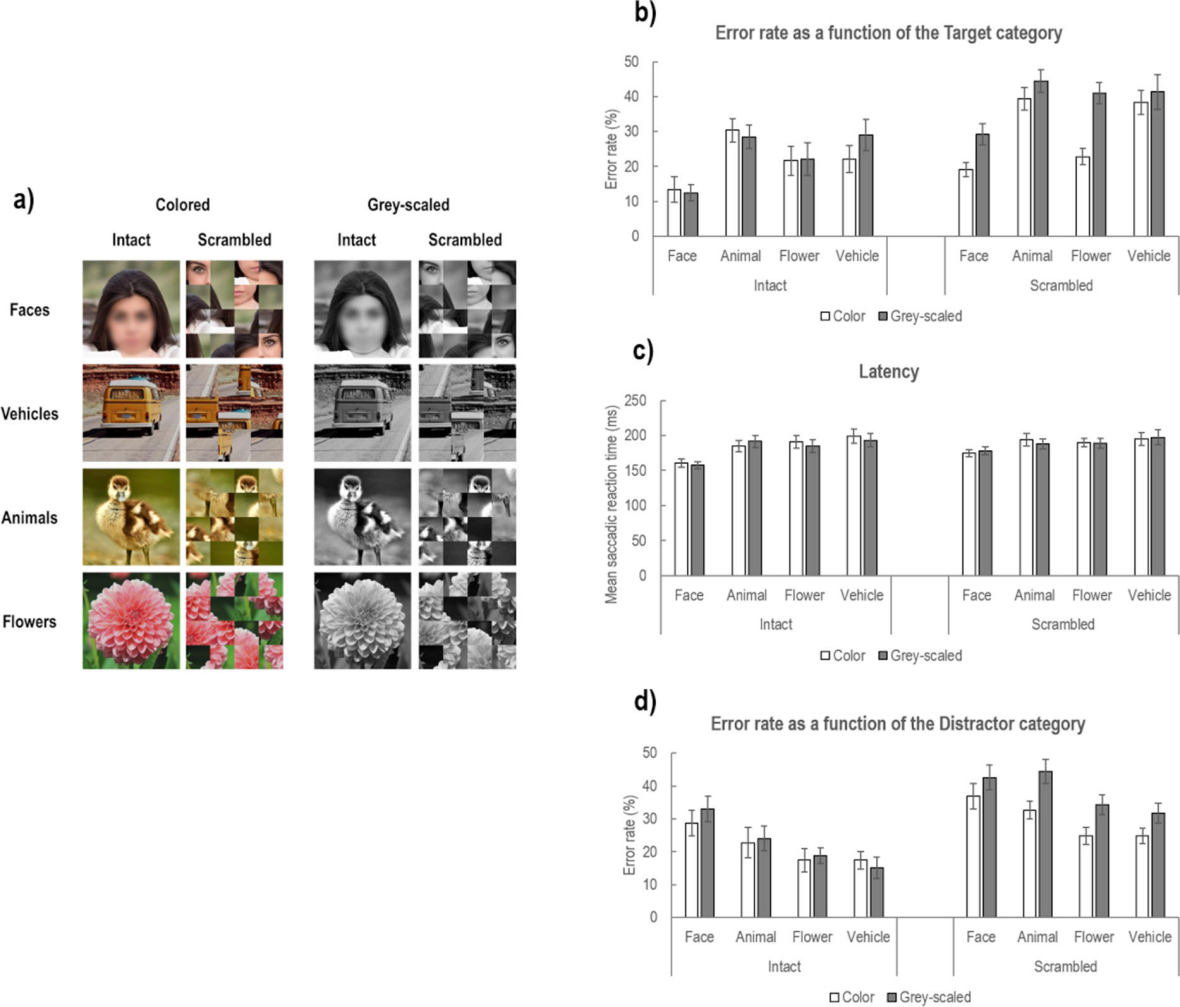
(a) Example of Intact and Scrambled stimuli used in Experiment 3 in their colored and gray-scaled versions. It should be noted that faces were blurred for publication purposes only. (b) Mean error rate (in % errors) and (c) latency or mean saccadic reaction times (in milliseconds) of saccades according to the Target category (Face, Animal, Flower, Vehicle), Scramble condition (Intact, Scrambled), and Color condition (color in light gray, gray-scaled in dark gray). (d) Mean error rate as a function of the Distractor category, as well as the Scramble and Color conditions. Error bars correspond to ± 1 *SE*. The images used in the experiment and in the figure are under the Pixabay license (https://pixabay.com), which allows free-from-copyright use and modification of images.

Stimuli were created from the same 60 colored images of face and vehicles used in Experiments 1 and 2, as well as 60 additional images downloaded from the “Pixabay” stock image base (https://pixabay.com/) under CC0 License. Half of these additional images contained animals while the other half contained flowers (Figure 9a). These images were cropped to a square format (1,000 × 1,000 pixels) and resized to 400 × 400 pixels, subtending 11.5 × 11.5° of visual angle at a viewing distance of 56 cm. This therefore resulted in a stimulus set of 120 images divided into four categories (Faces, Vehicles, Animals, Flowers). For each image, we created four versions (Figure 9a) according to two Scramble conditions (Intact and Scrambled using 16 thumbnails as in Experiment 2, in which local face configuration was more strongly altered) and two Color conditions (Colored and Gray-scaled). Mean luminance and RMS contrast of each image were equalized to match the mean luminance (0.44 for luminance values between 0 and 1) and RMS contrast (0.25) of the initial set of face and vehicle images used in Experiments 1 and 2 in order to allow comparisons with these experiments.

#### Procedure

Due to a change in the hosting institution of the experimenter, this experiment was not performed in the exact same conditions as Experiments 1 and 2. We, however, ensured that the viewing conditions remained comparable, namely in terms of visual angle of stimuli. Stimuli were displayed using the Psychtoolbox (Brainard, 1997; Pelli, 1997) implemented in MATLAB R2016a (MathWorks) against a gray background (luminance of 0.44) on a 21-in. CRT monitor with a spatial resolution of 1,280 × 1,024 pixels, a refresh rate of 75 Hz, and a mean gray luminance of 51 cd/m^2^. Participants were seated at a distance of 56 cm from the screen. Their head was maintained by a chin- and forehead rest. Eye movements were recorded with the same eye-tracker as in Experiments 1 and 2 using the same criteria to detect saccadic eye movements.

All participants underwent four experimental sessions during the experiment, one for each of the four image categories as target (Face, Vehicle, Animal, Flower). The order of the target categories was counterbalanced across participants. Within each session, the distractor image could be of the three remaining categories. The procedure and task were the same as in Experiments 1 and 2. For each session, a trial started with a white fixation cross subtending 0.73° of visual angle, displayed centrally for 800 to 1,600 ms (duration sampled from a uniform distribution) and followed by a gap (mean gray-level screen) of 200 ms. Following the gap, two images (a target and a distractor) appeared simultaneously on the left and the right of the display for 400 ms. The center of each image was lateralized at 8° from the center of the screen. The intertrial interval was fixed at 1,000 ms (see Figure 1b). Participants were instructed to make a saccade as fast as possible toward the target image. Each session was divided into four blocks of 30 trials according to the two Scramble conditions (Intact, Scrambled) and the two Color conditions (Colored, Gray-scaled) and the order of blocks was randomized. Within each block, the target appeared equally on the left and right visual fields, and the distractor belonged to the three remaining categories (10 trials per distractor category). Each image appeared once as target and once as distractor across the experiment. There were 120 trials per session (30 pairs of target-distractor images × 2 Color conditions × 2 Scramble conditions), resulting in a total of 480 trials in the whole experiment, which lasted about 25 min. Before the experiment, participants completed a training session comprising 16 trials in order to get familiarized with the task, using stimuli that were not subsequently used in the main experiment.

### Results

#### Data analysis

We analyzed the error rate (% error) and latency of correct saccades (in milliseconds) of the first saccadic response following stimulus appearance and meeting the same criteria as in Experiments 1 and 2. This resulted in removing 18% of the trials.

For each measure, the mean error rate and the mean SRT, we performed repeated-measures ANOVAs with the Target category (Face, Animal, Flower, Vehicle), the Scramble condition (Intact, Scrambled), and the Color condition (Colored, Gray-scaled) as within-subject factors. In order to assess the effect of the distractor category in driving error saccades, we additionally performed an ANOVA on mean error rate with the Distractor category (Face, Animal, Flower, Vehicle), the Scramble condition (Intact, Scrambled), and the Color condition (Colored, Gray-Scaled) as within-subject factors.

Effect sizes were estimated by calculating partial eta-squared (η_p_^2^). The significance level of tests was set at α = 0.05, and corrected *p*-values are reported for pairwise comparisons.

#### Error rate and latency of saccadic responses

The ANOVA performed on mean error rates (mER; Figure 9b) revealed a main effect of Target category (*F*_3, 45_ = 22.13, *p* < 0.0001, η_p_^2^ = .596). Pairwise comparisons revealed that participants made overall less errors when the target was a face (mean ± *SD*: 18.53 ± 8.76%) than when it was of another category (Animal: 35.66 ± 9.50%, *p* < 0.001; Flower: 26.90 ± 12.41%, *p* = 0.002; Vehicle: 32.67 ± 15.17%, *p* < 0.001). They also made less errors when the target was a flower than an animal (*p* = 0.01) or a vehicle (tendential, *p* = 0.07). There was also a main effect of the Color condition (*F*_1, 15_ = 23.49, *p* < 0.001, η_p_^2^ = .610) and of the Scramble condition (*F*_1, 15_ = 53.74, *p* < 0.0001, η_p_^2^ = .782), indicating that participants made overall more errors when stimuli were gray-scaled (31.00 ± 10.53%) than colored (25.88 ± 10.58%) and when they were scrambled (34.44 ± 9.21%) than intact (22.44 ± 12.27%). There was also a significant interaction between these three factors (*F*_3, 45_ = 5.42, *p* = 0.003, η_p_^2^ = .265). Further comparisons revealed that while the Color condition did not interact with the Target category when stimuli were intact (*p* = 0.41), but it did when stimuli were scrambled (*p* = 0.01). In particular, gray-scaling of scrambled images resulted in impairing performances when the target was a face (Colored: 19.05 ± 8.31%, Gray-scaled: 29.24 ± 12.24%, *p* = 0.01) and a flower (Colored: 22.75 ± 9.33%, Gray-scaled: 40.99 ± 12.51%, *p* < 0.0001) but not when it was an animal (Colored: 39.39 ± 12.75%, Gray-scaled: 44.42 ± 13.35%, *p* = 0.53) or a vehicle (Colored: 38.37 ± 13.87%, Gray-scaled: 41.31 ± 19.89%, *p* > 0.99).

The ANOVA performed on mean SRT (see Figure 9c) revealed a main effect of Target category (*F*_3, 45_ = 14.19, *p* < 0.0001, η_p_^2^ = .486). Pairwise comparisons showed that participants initiated their saccadic response toward the target faster when it was a face (168 ± 19 ms) than when it was of another category (Animal: 189 ± 30 ms, *p* < 0.001; Flower: 189 ± 29 ms, *p* < 0.001; Vehicle: 196 ± 38 ms, *p* = 0.001). However, there was no difference between mean SRT of responses toward animal, flowers, or vehicle targets (all *p*s > 0.99). There was neither a main effect of the Color (*F*_1, 15_ = 0.47, *p* = 0.50) nor of the Scramble condition (*F*_1, 15_ = 2.90, *p* = 0.11). However, the Scramble condition significantly interacted with the Target category (*F*_3, 45_ = 4.51, *p* = 0.007, η_p_^2^ = .231). Pairwise comparisons revealed that while face targets elicited faster saccadic responses than other target categories in both Scramble conditions, the mean latency of saccades toward face targets increased when stimuli were scrambled (177 ± 21 ms) than intact (159 ± 20 ms, *p* < 0.001), but scrambling of stimuli did not significantly impact the latency of saccades toward other target categories (all *p*s > 0.99).

#### Effect of the distractor category in eliciting error saccades

Given that, in Experiment 3, the distractor image could be of four categories (Face, Vehicle, Animal, Flower), we also analyzed the mER as a function of the distractor category (Figure 9d) in order to assess whether the higher error rate observed when the target was a vehicle (and the distractor was a face) in Experiments 1 and 2 actually reflected the higher attentional capture by face distractors, rather than a lower attractiveness of vehicle as targets. As in the analysis of mER as a function of the Target category, we found a main effect of the Color condition (*F*_1, 15_ = 14.22, *p* < 0.002, η_p_^2^ = .487), indicating that participants made more error saccades toward the distractor when stimuli were gray-scaled than colored, and a main effect of the Scramble condition (*F*_1, 15_ = 53.06, *p* < 0.0001, η_p_^2^ = .780), indicating that participants made more errors when stimuli were scrambled than intact. Importantly, we also observed a main effect of the Distractor category (*F*_3, 45_ = 24.90, *p* < 0.0001, η_p_^2^ = .624). Pairwise comparisons revealed that face distractors elicited more error saccades (35.26 ± 13.12%) than did flowers (23.84 ± 9.14%, *p* < 0.001) or vehicle distractors (22.24 ± 8.87%, *p* < 0.001). However, there was no significant difference between the proportion of error saccades elicited by face and animal distractors (30.48 ± 12.20 %, *p* > 0.99). Indeed, animal distractors also elicited more error saccades than did flower (*p* = 0.008) or vehicle distractors (*p* = 0.009). However, the Distractor category did not significantly interact with the other factors (all *p*s > 0.20), suggesting that although the error rate was overall increased when stimuli were gray-scaled or scrambled, the effect of the Distractor category remained similar in these conditions.

Overall, these results suggest that both face and animals acted as more powerful distractors than flowers and vehicles, even when holistic processing was altered via scrambling. In order to disambiguate which face or animal distractors would take over when images from the two categories are displayed within the same trial as target or distractor, we performed a paired *t* test between the conditions in which (a) the target was a face and the distractor was an animal and (b) the target was an animal and the distractor for each of the Color and Scramble conditions. Results revealed that participants made more error saccades toward face distractors when the target was an animal than they did toward animal distractors when the target was a face irrespective of the Color or Scramble condition (Color-Intact: Animal target/Face distractor = 46.88 ± 22.01%, Face target/Animal distractor = 20.63 ± 17.09%, *t*_15_ = 5.80, *p* < 0.001; Color-Scrambled: Animal target/Face distractor = 50.79 ± 20.51%, Face target/Animal distractor = 30.23 ± 14.92%, *t*_15_ = 4.70, *p* < 0.001; Gray-scaled-Intact: Animal target/Face distractor = 46.66 ± 23.43%, Face target/Animal distractor = 14.39 ± 13.78%, *t*_15_ = 4.72, *p* < 0.001; Gray-scaled-Scrambled: Animal target/Face distractor = 58.03 ± 20.27%, Face target/Animal distractor = 38.90 ± 17.90%, *t*_15_ = 2.68, *p* = 0.02), suggesting that face acted as more powerful distractors than animals did.

### Discussion of experiment 3

Experiment 3 aimed at further assessing the role of features (color, or shapes linked to a high within-category structural homogeneity) and degree of animacy, characteristic of faces in driving the ultra-rapid and involuntary responses toward them in intact and scrambled images by adding target categories sharing similar attributes with faces and manipulating the color content of stimuli. In agreement with previous findings (Guyader et al., 2017), we found that gray-scaling of stimuli had no significant effect on the latency of saccades toward the different target categories, suggesting that the ultra-rapid saccades toward intact or scrambled faces are not driven by their color content. Furthermore, although scrambling of stimuli resulted in increasing the latency of saccadic responses toward face targets, faces always elicited faster saccades than any other target categories, with no significant difference in mean SRT between these categories. This therefore goes against the idea that (a) attributes linked to the high structural homogeneity of faces such as their roundness (which also characterized flower targets) or (b) higher-level attributes such as the degree of animacy (which also characterized animal targets) facilitated the ultra-rapid saccades toward faces since no bias was observed for the flowers and animal targets in terms of mean SRT.

The analysis of mean error rate as a function of the Target category, however, revealed that participants made overall more errors when stimuli were gray-scaled than in color, suggesting that gray-scaling of stimuli generally impairs the accurate detection of the target. Furthermore, scrambled faces targets were categorized with less errors than the other target categories, and scrambled flower targets were also categorized more accurately than animal and vehicle targets and were more strongly impacted by gray-scaling of stimuli than the other target categories. Since flowers and faces shared a high degree of structural homogeneity characterized by a round shape and curved lines, it can be assumed that such attributes, as well as their color content, are also used to facilitate their detection.

Finally, Experiment 3 also allowed us to assess the effect of the distractor category in driving error saccades. We found that faces acted as more powerful distractors than the other distractor categories, irrespective of the Scramble or Color condition, supporting the idea that in Experiment 2, the higher rate of error saccades toward face distractors when stimuli were scrambled could not simply be attributed to a lower attractivity of vehicle targets. Interestingly, we also observed that animals acted as more powerful distractors than did flowers and vehicles. This result has been previously observed (Boucart et al., 2016; Crouzet et al., 2010; Guyader et al., 2017) and is consistent with previous findings that biological/animate stimuli tend to capture attention more than inanimate objects (New, Cosmides, & Tooby, 2007) but may also be explained by the fact that animals do have a face and therefore salient face parts that may capture attention.

It is interesting to note that results in terms of error rate as a function of the Distractor category in Experiment 3 did not necessarily mirror results in terms of error rates as a function of the Target category. For example, while animals acted as powerful distractors, they were not accurately detected as targets. On the other hand, flowers were detected more accurately as target but were not powerful distractors. Finally, faces were both accurately detected and powerful distractors while vehicles were neither. These differences could be explained by the fact that faces and flowers share a high degree of within-category homogeneity (e.g., round shape), which could make them easier to search for and detect in scrambled stimuli. However, flowers may not contain isolated features salient enough to capture attention as faces do. On the contrary, the animal and vehicle categories were characterized by a higher variability, which may make them more difficult to detect. Yet, animals contain salient features (e.g., eyes or face-like parts) that may capture attention as faces do in contrast to flowers and vehicles. Importantly, this dissociation also suggests that error rates as a function of the target or the distractor categories reflects different mechanisms underlying the task and supports the distinction made throughout our experiments between (a) error rates as a function of the target indexing the ability to accurately detect the target and (b) error rates as a function of the distractor indexing its attentional capture.

## General discussion

The present study aimed to investigate the extent to which ultra-rapid and involuntary saccades oriented toward face stimuli during a saccadic choice task could be supported by their holistic processing. First, our results replicated previous findings indicating that in comparison to other categories of visual stimuli such as vehicles, faces elicit much faster and more involuntary saccades toward them (Crouzet et al., 2010; Guyader et al., 2017; Kauffmann et al., 2019). Critically, while disruption of holistic processing slightly impaired the ability to accurately categorize faces (Experiment 1), it only had a limited impact on the speed of saccadic responses toward them and did not affect their attentional capture, even when the spatial configuration and low-level properties of stimuli were further altered via a scrambling procedure (Experiment 2) or when the role of attributes such as color were controlled for (Experiment 3). Our results rather indicate that information contained in isolated face features is sufficient to elicit fast and automatic orienting responses toward faces. The significance of these results and their integration within the theoretical frameworks of face processing are discussed in the following.

### Holistic processing does not account for the involuntary orienting response toward faces but may partly support their ultra-rapid detection

In Experiment 1, we investigated the role of holistic processing of faces during the saccadic choice task by manipulating the orientation of stimuli that were either upright or inverted, the latter condition being used to disrupt holistic processing. We observed that while participants made more errors when the target was a vehicle than a face (i.e., more involuntary saccades toward face than vehicle distractors) in both orientation conditions, this effect was reduced when stimuli were inverted, due to an increase in error rate in the Inverted relative to the Upright condition when the target was a face. However, the proportion of error saccades toward face distractors (i.e., when the target was a vehicle) was not influenced by the Orientation condition. Experiments 2 and 3 further supported this finding by showing that face distractors elicited more error saccades than did other categories of distractors and that this effect was not reduced by the progressive alteration of local face configuration and their amplitude spectrum in Experiment 2 or by gray-scaling of stimuli in Experiment 3. This last result therefore suggests that holistic processing does not mediate the involuntary orienting response toward faces previously observed during a saccadic choice task (Crouzet et al., 2010; Guyader et al., 2017; Kauffmann et al., 2019). This finding is consistent with a study by Bindemann and Burton (2008) reporting an attentional bias for upright but also inverted faces, each type of stimuli equally drawing attention. It, however, contradicts previous findings of Gilchrist and Proske (2006), who used an antisaccade task in which participants were instructed to perform a saccade away from upright or inverted peripheral faces. These authors found that participants made more errors (i.e., more involuntary prosaccade toward the faces) when the faces were upright than inverted, suggesting that it is more difficult to inhibit saccadic responses toward upright than inverted faces. Other studies also suggested that holistic processing plays a role in the attentional capture by faces. Indeed, it has been shown that, even when irrelevant to the task at hand, the mere presence of a face distractor captures attention and can interfere with the task (Ariga & Arihara, 2018; Langton et al., 2008; Sato & Kawahara, 2015; Theeuwes & Van der Stigchel, 2006). However, this effect was found to be attenuated when faces are inverted (Devue et al., 2012; Langton et al., 2008). Yet, in these studies, inverted face distractors still captured attention more than other types of distractors. Overall, accuracy results across our three experiments, along with results from previous studies, suggest that, irrespective of their spatial configuration, faces capture attention more than other types of visual stimuli.

Similarly, evidence that holistic processing accounts for the ultra-rapid detection of faces as measured by saccadic reaction times in the present study was scarce. On one hand, we observed that, on average, stimulus inversion (Experiment 1) only had a marginal effect on mean saccadic reaction times toward target images (3 ms increase relative to the Upright condition) and that further alteration of their low-level properties and spatial configuration via scrambling in Experiments 2 and 3 resulted in a ∼10-ms increase in latencies relative to their intact version. However, in all experiments, faster saccades elicited much faster saccadic responses than did other target categories. Critically, the minimum saccadic times toward face targets remained extremely and consistently short across all experimental conditions (minSRT of 120–130 ms) of Experiments 1 and 2, suggesting that faces elicited ultra-rapid saccades even when their low-level properties and spatial configuration were strongly altered. Previous studies investigated the speed of face detection using visual search tasks, in which a face target was to be detected among a set of distractor images with various set sizes (e.g., Hershler & Hochstein, 2005; VanRullen, 2006). These studies also manipulated the holistic processing of stimuli through inversion (VanRullen, 2006) or scrambling (Hershler & Hochstein, 2005). In agreement with the present results, stimuli inversion only had a limited impact on the visual search for faces, which was still very efficient. However, the scrambling of stimuli strongly impaired detection performances in Hershler and Hochstein's (2005) study, while this was not the case in our experiment. This discrepancy may be due to the fact that our study only involved one distractor (vs. up to 65 in Hershler & Hochstein, 2005), which may have rendered the target detection easier. In summary, while alteration of spatial configuration of faces in the present study resulted in generally impairing the speed their detection, it did not reduce the bias in favor of face targets for which saccadic reaction times were always much faster than for vehicle targets. This result indicates the ultra-rapid detection of faces may be partly supported by their holistic processing but that, although reduced, this bias still persists when holistic processing is disrupted.

### What drives ultra-rapid and involuntary saccades toward faces?

In order to assess whether the ultra-rapid and involuntary saccades toward faces can be mediated by the fast detection of specific and salient face features, we examined the endpoints of saccades. Results of these analyses in Experiment 1 revealed that participants consistently oriented their gaze toward the eye region in face targets, whether they were upright or inverted. This pattern is consistent with many studies showing that the eye region in faces is one of the first and most fixated region during free viewing (e.g., Bindemann et al., 2009; Coutrot & Johnston, 2016) and would be particularly relevant for tasks such as face detection but also gender, emotion, or face recognition (Blais, Jack, Scheepers, Fiset, & Caldara, 2008; Hsiao & Cottrell, 2008; Lewis & Edmonds, 2003; Peterson & Eckstein, 2012; Schyns, Bonnar, & Gosselin, 2002; Vinette, Gosselin, & Schyns, 2004). At first sight, this pattern of results would thus support the idea that rapid saccades toward faces can be elicited by the mere detection of an eye, irrespective of face configuration. However, this pattern was not replicated in Experiment 2, in which stimuli were scrambled and face parts such as the eyes were randomly relocated within the images.

The discrepancy between the two experiment might have alternative explanations. First, as previously suggested, it is possible that the bias toward the eye region observed in Experiment 1 actually reflects a “center-of-gravity” bias. Indeed, previous studies have shown that during free viewing or visual search tasks, the first fixations are frequently located near the center of gravity of the target configuration (Findlay, 1982), which usually corresponds to the eye region in front and mid-profile faces (Bindemann et al., 2009) as those used in our experiments. The absence of such pattern in Experiment 2 could thus be due to the fact that the progressive alteration of stimuli's spatial configuration resulted in abolishing this bias. Alternatively, the difference between the two experiments could be attributed to the fact that while the location of the eye region in faces in Experiment 1 was quite consistent across face images and could be easily predicted in the context of upright and inverted faces, this was not the case in Experiment 2. Thus, the difference in the observed pattern of results would rather reflect a difference in strategy for saccade programming. This explanation would be in line with previous studies by de Haas and colleagues showing that our face-processing system is tuned to process face features at their typical location (de Haas & Schwarzkopf, 2018; de Haas et al., 2016). It is also supported by the pattern of saccadic endpoints observed in Experiment 2, which were located quite consistently in the same part of the stimuli in each Scramble condition (upper part of the image in the 2-thumbnail condition and inner lateral part of the image in the 9- and 16-thumbnail conditions), irrespective of the face content available at these locations. Overall, the analyses of saccadic endpoints indicate that saccadic responses may be preferentially programmed toward the eyes in faces when their location can be predicted but not when they are at an atypical location.

This leaves us with the following question: *what* drives ultra-rapid and involuntary saccades toward faces? Results of Experiment 3 allowed us to rule out the role of attributes such as color, but overall, the present study was not designed to fully disambiguate this question. Indeed, the fact that the eyes were not necessarily targeted in scrambled stimuli does not preclude that they (or that other face parts) are used for the ultra-rapid detection and attentional capture by faces in inverted or scrambled stimuli. Furthermore, the fact that animals acted as almost as powerful distractors as faces may suggest that their degree of animacy or the fact that they both contain face features that were preserved in scrambled stimuli plays a role in their attentional capture. Regarding the speed of face detection, previous studies suggested that a likely contributing factor is the rapid extraction of low-level features of faces such as their low spatial frequency content (Goffaux et al., 2010; Goffaux & Rossion, 2006; Guyader et al., 2017; Petras et al., 2019) or amplitude spectrum information (Crouzet & Thorpe, 2011; Honey et al., 2008; Vanrullen, 2006). For example, Crouzet and Thorpe (2011) used a saccadic choice task in which stimuli were built by combining the amplitude spectrum of a face (i.e., mean distribution of energy across orientations) with the phase spectrum of a car (i.e., spatial relations within the image) and vice versa. They showed that this manipulation resulted in significantly reducing the bias for face stimuli, indicating that information from amplitude spectrum of faces is used to elicit fast orienting responses toward them. Similarly, Honey et al. (2008) showed that even when no phase information was available in stimuli and only amplitude spectrum information was shown (i.e., phase-scrambled stimuli), the fastest saccadic responses were directed more often toward the stimulus containing the amplitude spectrum of a face. Yet, an explanation for fast orienting responses toward faces simply in terms of their low-level properties is not supported by results of Experiment 2 of the present study, in which we found that minSRT toward scrambled face targets were comparable to those observed for intact face targets in Experiment 1, even though our scrambling procedure resulted in a strong alteration of stimuli amplitude spectra. Our results therefore suggest that the information available in isolated face features may also be sufficient and salient enough to elicit fast and involuntary orienting responses toward them. Overall, past findings as well as results of the present study indicate that the ultra-rapid orienting responses toward faces may not be solely based on the processing of one specific visual information but may rather rely on the extraction of a combination of features. Further studies manipulating more systematically the availability of low-level (e.g., amplitude spectrum) and higher-level face features (e.g., face parts) within the same experiment would be necessary to better understand their relative role in driving ultra-rapid and involuntary saccade toward faces.

### Holistic representations and attributes such as color or shape would be used as “search templates” to accurately detect faces

While we argue against a purely holistic processing account to explain the speed of saccades and the attentional capture by faces, our results, however, suggest that holistic processing does play a role in facilitating the detection and categorization of faces. Indeed, Experiment 1 revealed that the accuracy of saccades toward face targets was reduced when holistic processing was disrupted via stimulus inversion. This was also supported by the overall increase in error rate for face targets observed when holistic processing was further disrupted by the scrambling of stimuli in Experiment 2 (mean error rate for the face Target condition: 15%) and Experiment 3 (mean error rate for the face Target condition: 19%) in comparison to the conditions in which faces were intact (mean error rate: ∼10%), as well as by the reduced mean latency of saccades toward face targets when stimuli were scrambled in Experiment 3. This finding is consistent with previous studies showing that disruption of holistic processing impairs their detection during visual search (Hershler & Hochstein, 2005; Lewis & Edmonds, 2003; Rousselet, Macé, & Fabre-Thorpe, 2003). In the context of visual search, it has been proposed that when searching for a target among distractors, a “search template” of target information would be used to prioritize and facilitate the processing of information matching this template in order to increase search efficiency (Desimone & Duncan, 1995). Under this assumption, the lower accuracy observed to detect face targets when holistic processing is disrupted would indicate that holistic representations are used as search template for faces. Results of Experiment 3 additionally suggest that the search template for faces may also include attributes characteristic of faces such as their color or shapes as the accuracy of saccades toward face targets was reduced when stimuli were gray-scaled and as the detection of targets sharing a similar shape (i.e., flowers) was facilitated.

### Conclusion

Faces constitute a special class of stimuli that immediately attract the gaze and capture attention but also differ from other categories of visual stimuli in that they are processed holistically. The present study, however, revealed that a holistic processing is not a prerequisite to such ultra-rapid and involuntary orienting responses toward faces, which may rather be driven by the extraction of information available in isolated face parts, irrespective of their configuration—although no specific face feature could be precisely identified. Our results, however, support the idea that when searching for a face, holistic representations and attributes such as color and shapes (e.g., curved lines) would be used as a search template to facilitate its correct detection.

## Footnotes

1 It should be noted that these images were not the ones used in previous studies (e.g., Crouzet et al., 2010; Guyader et al., 2017; Kauffmann et al., 2019). This was motivated by the fact that we wanted to make our stimuli openly available but could not obtain the necessary information about the copyright of the image database used in previous studies. We therefore built this new stimulus set using a free from copyright image base in order to ensure they could be shared in open access.
